# Applying NV center-based quantum sensing to study intracellular free radical response upon viral infections

**DOI:** 10.1016/j.redox.2022.102279

**Published:** 2022-03-18

**Authors:** Kaiqi Wu, Thea A. Vedelaar, Viraj G. Damle, Aryan Morita, Julie Mougnaud, Claudia Reyes San Martin, Yue Zhang, Denise P.I. van der Pol, Heidi Ende-Metselaar, Izabela Rodenhuis-Zybert, Romana Schirhagl

**Affiliations:** a Department of Biomedical Engineering, University of Groningen, University Medical Center Groningen, Groningen, 9713AV, the Netherlands; b Department of Dental Biomedical Sciences, Faculty of Dentistry, Universitas Gadjah Mada, Jalan Denta 1, Yogyakarta, 55281, Indonesia; c The University of Poitiers, Bât. B2, 2 rue Charles-Claude Chenou TSA 51106, F-86073, Poitiers, Cédex 9, France; d Department of Medical Microbiology and Infection Prevention, University of Groningen, University Medical Center, Groningen, 9713AV, the Netherlands

**Keywords:** Fluorescent nanodiamonds, Free radicals, Viral infections, Diamond magnetometry, NV centers, ROS

## Abstract

Although viruses are known to modify the free radical concentration in infected cells, the exact location and concentrations of such changes remain unknown. Although this information is important to understand the virus pathogenesis and design better anti-viral drugs or vaccines, obtaining it with the conventional free radical/ROS detection techniques is impossible. Here, we elucidate the utility of diamond magnetometry for studying the free radical response of baby hamster kidney-21 cells upon Semliki Forest virus infection. Specifically, we optically probe the alterations in free radical concentration near infectious viruses via measuring the spin–lattice relaxation (T_1_) of NV defect ensembles embedded in intracellular nanodiamonds. We performed measurements both at random locations as well as close to the virus entry by conjugating viruses to nanodiamond sensors. We observed alterations of T_1_, which represent the intracellular free radical concentration during the viral replication process. Moreover, relaxometry is also used to monitor real-time free radical variation during the early infectious process.

## 1 Introduction

The pandemic of SARS-CoV-2 has once again highlighted the importance of virology research as it claimed millions of lives, affected billions of people and brought almost the entire world to a standstill. Redox imbalance in viral infections is an extensively studied topic. Most studies on oxidative stress in viral infections do not differentiate between paramagnetic and non-paramagnetic reactive molecules. Instead they assess the amount of the reactive molecules in cells or evaluate their overall molecular and enzymatic response to probe the infection induced redox imbalance. However, this approach is not always useful and sometimes type/nature of an individual reactive species needs to be considered. For instance, although the amount of oxygen radicals upon influenza virus infection has been shown to increase [1], similar effects for hydroxyl radicals have not been reported [2]. Another example would be the non-universal anti-viral activity of nitric oxide which changes depending on the virus it is interacting with [2]. Free radicals play a key-role during infection, virus mutation and disease pathogenesis [[1], [2], [3], [4], [5], [6], [7], [8]]. However, they are extremely short lived [9] and are generated in small concentrations, which complicates studying them. Therefore, a method capable of real-time detection of the variation in free radical amount with high spatial resolution can significantly boost virology research.

Conventionally, fluorescence-based assays [10,11] or UV-VIS absorption spectroscopy techniques [12] utilizing a wide array of molecular probes are deployed to measure the amount of intracellular free radicals. Here, the amount of fluorescence or absorbance is directly correlated with the concentration of free radicals. In all such methods, conducting real-time, kinetic studies is impossible due to bleaching and irreversible reactions between the probes and the free radicals. Another approach is detecting the expression of genes encoding enzymes involved in coping with the oxidative stress (such as superoxide dismutase or catalase) using quantitative polymerase chain reactions (qPCR) [13]. Although this method can be specific for certain radicals, it does not provide any information on the spatial and temporal evolution of the free radical response. Electron paramagnetic resonance (EPR) and magnetic resonance imaging (MRI), methods routinely used for studying free radicals in chemistry are limited in sensitivity. This is especially crucial when high spatial resolution is required or only limited amounts of sample are available (inside of a cell for example).

Here, we demonstrate that relaxometry can be an excellent technique to study intracellular free radicals upon viral infection. In free radical detection, this method offers distinct advantages in terms of biocompatibility, sensitivity [14], resolution, relatively low-cost equipment and lack of complex chemistries. Most importantly, internalized fluorescent nanodiamonds (FNDs) do not get consumed or bleached in a reaction, which makes real-time long-duration measurements possible. Moreover, this is the only method that simultaneously detects the real-time impact of viral infection on cytoplasmic transport via tracking the nanodiamond movement inside the cell. In this method, an optical readout of the magnetic environment can be acquired using the quantum sensing properties of the Nitrogen- Vacancy (NV) defect center in a diamond crystal [[15], [16], [17]]. Hence relaxometry has a great potential to optically investigate the amount of intracellular free radicals. Unpaired electrons of free radicals offer magnetic noise to the NV centers embedded in FNDs ingested by cells. In fact, detecting free radical generation in wild-type and mutant yeast has recently been achieved [18].

Here, we optically probe the free radical response of host baby hamster kidney-21 (BHK-21) cells upon Semliki Forest virus (SFV) infection by measuring the relaxation time (T_1_) of NV ensembles embedded in an intracellular FND conjugated to SFV as well as bare FNDs. Moreover, the location and duration of host cellular radical response during viral infection, and the impact of viral infection on the cytoplasmic transport is elucidated for the first time.

## 2 Materials and methods

### 2.1 Relaxometry experiments to measure T_1_ relaxation

#### 2.1.1 Relaxometry setup

**Fig. 1:**
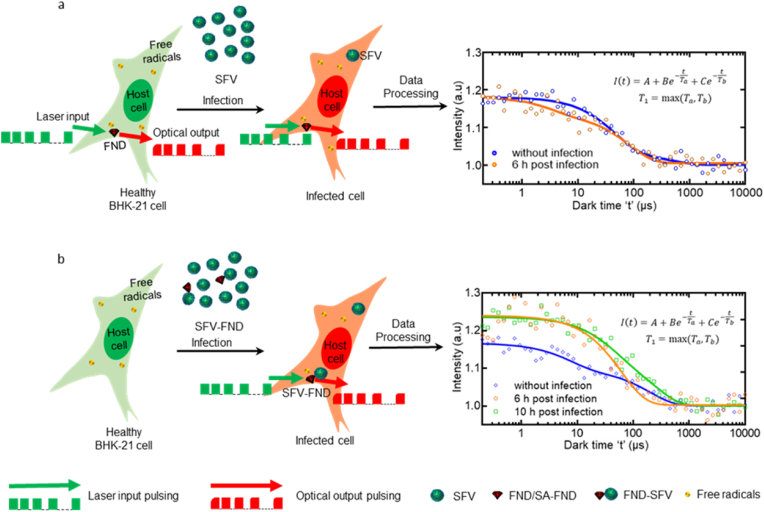
Schematic representation of probing general intracellular free radical response (a), and free radical response near viral particles (b) of BHK-21 upon SFV infection using relaxometry.

To perform relaxometry, we utilized a home-made magnetometry setup. The setup is in principle a confocal microscope with a few changes as described below. First, we implemented an acousto-optical modulator (Gooch & Housego, model 3350-199) to conduct the pulsing sequence shown in Fig. 1. For focusing and light collection we used a 100x magnification oil-immersion objective (Olympus, UPLSAPO 100XO). 50 μW laser power measured on top of the objective lens was the optimal laser power for avoiding damage to the cells while being high enough to polarize the NV centers. The photons emitted by an FND are detected using an avalanche photodiode (Excelitas, SPCM-AQRH) after passing through a 600 nm long-pass filter.

#### 2.1.2 Selecting an intracellular FND for T_1_ measurements

Using a bright field camera (Thorlabs) we selected FND particles (Adámas Nanotechnologies, Inc., USA) well inside a cell with a brightness >2 million photon counts/s. These nanodiamonds are very well characterized in the literature [[21], [22], [23]]. To avoid artifacts, we made sure, that there were no significant differences between the count rates of the different experimental groups. Such bright particles are clearly visible on background fluorescence originating from the biological material. This is different from commonly used diamond crystals that contain single NV defects [19,20]. While properties of selected NV centers are superb, there is a large variation in single defect quality and thus usually a preselection is needed. This approach is not practical for measurements in a biological system since we cannot reuse a particle as a sample is typically discarded after every experiment. Most importantly this would also make it impossible to compare different experiments on different cells and different particles. Hence FND containing ensembles of ∼500 NV centers are used in this work. Thus, we always measure the sum of responses of many different NV centers in our measurements, which makes measurements more robust and repeatable.

#### 2.1.3 Experimental protocol

To experimentally determine the T_1_, we use the spin relaxometry protocol developed by Tetienne et al. [20]. In short, during this sequence we pump the NV centers to the bright state and probe after different dark times if it is still there. The biggest advantage of this sequence is that it does not require microwaves. In biological samples, the water in the cell culture medium absorbs the microwaves. This not only deteriorates the signal but also leads to thermal dissipation. During a T_1_ measurement we first polarize the NV centers ensemble in the 70 nm FND ingested by the BHK-21 cells using 5 μs pulse of a 532 nm laser. Then, we turn the laser off for a predefined time interval, known as dark time, which varies between 0.2 μs to 10^6^ μs. The laser pulse sequence used in this work for exciting the NV ensemble is shown in Fig. 1 (green). After exciting the NV ensemble, photons emitted from an FND are collected using a photodetector. The number of photos hitting the detector is plotted against the dark time as shown in Fig. 1 (red). Each individual measurement takes some hundreds of microseconds. However, to improve the signal to noise ratio we repeated the measurement 10,000 or 85,000 times depending on the experiment. To record the location and to avoid losing the particle we automatically pause every 5 s to track and recenter the particle. The entire sequence takes around 10 min for 10,000 and 85 min for 85,000 repetitions.

#### 2.1.4 Data analysis

Once the data acquisition is complete, the data was processed further to quantify the T_1_. The model we used to fit the T_1_ data was established earlier [21] and is described by

PL(t) = I_inf_ + C_a_ e^-t/Ta^ + C_b_ e^-t/Tb^ where T_1_ = max (Ta, Tb)

Technically there are hundreds of NV centers in every particle. To simplify the fitting procedure the relaxation time of the ensemble is approximated to have two components: the NV centers with shorter (Ta, closer to the surface or in a more disturbed environment) and one with longer T_1_ ((Tb) deeper in the crystal or less perturbed) [22]. Between the two constants, the longer T_1_ time was selected for quantification because it is more sensitive to changes of the NVs' surrounding. This model and some of the experimental parameters was determined by Perona Martinez et al. [21] by testing the model's ability to determine known concentration in a controlled environment.

### 2.2 Cell and virus model

BHK-21 cells were maintained in Roswell Park Memorial Institute (RPMI)-1640 medium (Life Technologies, Breda, The Netherlands) supplemented with 10% fetal bovine serum (FBS), 1% Penicillin/streptomycin and 1% Glutamax at 37 °C and 5% CO_2_. All the culture medium components were purchased from Gibco, Life Technologies, the Netherlands. We used active SFV (clone SFV4 produced as in Ref. [23]) and inactivated viruses. Inactivated SFV was obtained by UV irradiating (Miller) the SFV stock solution for 2 h. The inactivation was confirmed using a TCID_50_ assay where the virus titer was found to be below the detection limit of the assay.

To prepare the fluorescently-labeled virus, sucrose purified SFV (TCID_50_ titer: 2.8 × 10^10^ TCID_50_/mL) was labeled with 3,3′-Dioctadecyloxacarbocyanine perchlorate (DiO) (Vybrant, Thermo Fisher Scientific, the Netherlands) following the protocol described by Hoornweg et al. [24]. Specifically, 25 μl of the SFV stock solution were mixed with 2 μL 1 mM of DiO cell-labeling solution in a final volume of 50 μL. After 10 min of incubation at room temperature in the dark, the unattached dye was removed by a Sephadex G-50 Fine column (Pharmacia). HNE buffer (5 mM HEPES, 150 mM NaCl, 0.1 mM EDTA, pH 7.4) was used to equilibrate the column. DiO-labeled virus was stored at 4 °C and used within 2 days. The effect of DiO labeling on the virus titer was determined using the fifty-percent tissue culture infective dose (TCID_50_) assay where the titer was found to be 2 × 10^8^ TCID_50_/mL post labeling. Individual virus particles labeled with DiO were further characterized by fluorescence microscopy (Delta Vision Elite). For the microscopic analysis, 100 μl of 100-times diluted DiO-labeled SFV (DiO-SFV) solution were added to one quarter of the glass bottom Petri dish and imaged with a 488 nm laser. Emission was collected by a 100 × oil immersion objective with a numerical aperture of 1.40 and imaged through a FITC filter and a charge-coupled-device camera. Image analysis (intensity-particle number) was carried out by using Particles Analyzer plugin of ImageJ (Fiji, fiji.sc/) as shown in Fig. S1a.

### 2.3 SFV-FND conjugates

The biotin-streptavidin technique was used to achieve SFV conjugation with FNDs because of its specificity and robustness [[25], [26], [27], [28]]. Specifically, 50 μL of SFV stock solution were mixed with 1 μL freshly prepared 5 mM of Sulfo–NHS–LC-Biotin (EZ-Link, ThermoFisher Scientific) solution for 2 h at room temperature. Unbound biotinylation agent was removed by micro-dialysis (3.5K MWCO, Pierce, Thermo Scientific) against HNE buffer for 4 h at room temperature. Subsequently, 10 μL biotinylated SFV (TCID_50_ titer: 3 × 10^7^ TCID_50_/mL) were added to 10 μL SA-FND (100 nm, 1 mg/mL in PBS, Adámas Nanotechnologies, Inc., USA), then the mixture was incubated at 25 °C for 2 h. The SFV-FND conjugates were used for further experiments without further purification. To examine the conjugating efficiency of SA-FND with biotinylated SFV, the SFV was firstly modified by biotin, then labeled by DiO. The resulting DiO labeled biotinylated SFV was then conjugated to SA-FND using the same protocol. After this, the mixture (DiO-SFV-FND) was diluted 10 times by PBS and examined by fluorescent microscope (DeltaVision Elite) through FITC and A594 filters, followed by image analysis using FIJI.

### 2.4 Virus titer quantification

The virus titer of the SFV (infectious, inactivated, fluorescent-labeled) stock solution was determined using the TCID_50_ assay. TCID_50_ is the dilution of the virus stock required to infect 50% of the given cell population. In this assay, 40,000 cells plated in flat-bottom 96 well plates (Corning Costar) were infected with 10^−1^ through 10^−12^ dilution of SFV stock solution made with 2% (FBS) RPMI medium. At 1-h post infection (hpi), 150 μl of 10% (FBS) medium was added over the cells. Every 24 h until 72 hpi, the cells were examined for signs of SFV induced cytopathological effects (CPE) using a light microscope (Leica DMIL LED). The numbers of live and dead wells were counted and further used to calculate the TCID_50_ of the initial virus stock solution by the Spearman-Kärber algorithm given by Hierholzer and Killington [29].

### 2.5 Measuring free radical response of BHK-21 cells to SFV infection at discrete timepoints

The general intracellular free radical response of BHK-21 cells was measured by sequentially incubating FND and SFV with host cells. One day before the experiment, 1 μg/ml 70 nm FND suspension (10% FBS + 90% RPMI-1640 medium) was added to 20,000 cells in one quarter of a glass bottom Petri dish (Greiner bio-one, Germany). The FND suspension was made using a protocol established in our group [19,20]. Then host cells having intracellular FNDs were infected with SFV with a multiplicity of infection (MOI = number of virus particles added per cell) of 20. Specifically, cells were covered with 100 μL infection medium (2% FBS + 98% RPMI-1640 medium + virus particles). We made five such dishes for every experiment. In addition, we prepared another Petri dish where cells were treated with medium alone without any virus particles. This was treated as a control sample. We used three experimental groups where cells were treated with (i) infectious SFV, (ii) UV-inactivated SFV and (iii) plain medium at the 0 h timepoint.

To investigate the intracellular free radical response near the viral particles, virus-diamond conjugates (SFV-FND) were used to infect host cells. 40,000 BHK-21 plated in a four-quartered glass-bottom Petri dish (Greiner bio-one, Germany) were incubated with SFV-FND with MOI = 20. In this experiment, SA-FND without viral particles treated BHK-21 cells was set as a control group. Here three experimental groups were cells treated with (i) infectious SFV-FND, (ii) inactivated SFV-FND and (iii) SA-FND at the 0 h time point.

At 1 hpi, the infection medium was removed, and cells were supplemented with 400 μl of 10% medium. After this, cells were maintained in the incubator except during the treatment or the T_1_ measurement. At every timepoint (control, 2, 4, 6, 8, 10 hpi), we removed one Petri dish from the incubator and measured T_1_ relaxation on four different FNDs ingested by four different cells in the same Petri dish. Three measurements on a single Petri dish were completed within a maximum of 1 h after removing it from the incubator. Such an experiment was repeated three independent times per group.

### 2.6 Investigating pseudo real-time variation in intracellular free radical amount post SFV infection

The real-time variation of intracellular free radicals near viral particles was investigated by two steps. First, seeded BHK-21 cells were incubated with 100 μL SFV-FND (MOI = 20, SFV equiv.) for 45 min at 4 °C. Later, unattached SFV-FND were removed by washing the cells three times with cold RPMI. BHK-21 cells were kept on an ice bath with cold cell culture medium until further experiments. Then we applied the T_1_ measurement cycle for 85,000 times in 85 min using the same particle (as described in **Section S10**). For comparison, a rolling window experiment was also performed on the inactive SFV-FND conjugates or only SA-FND attached BHK-21 cells. Each experimental group was repeated 9 times.

The data were analyzed using a “moving window algorithm” to determine the temporal evolution of T_1_ in healthy and treated cells. Details of the algorithm are explained in section 3.8. The output of the algorithm is smoothened using GraphPad Prism. (GraphPad Software, San Diego, California USA, www.graphpad.com).

### 2.7 Imaging virus entry into the cell

To investigate how much time is required for SFV and SFV-FND to enter the cells, the virus-tracking experiments were performed. Briefly, BHK-21 cells were seeded one day before the experiment. Next day, cells were washed twice with cold phenol-red RPMI. Glucose oxidase (GLOX) was added to prevent photo toxicity as elaborated in the literature [30,31]. Subsequently, 100 μL DiO- SFV or DiO-SFV-FND (∼10^7^ TCID_50_/ml SFV equiv.) was added to BHK-21 cells and cells were incubated for 45 min at 4 °C. Later, unattached virus or conjugates were removed by washing the cells three times with cold RPMI. Subsequently, the temperature was rapidly elevated to 37 °C using a thermostatic stage. This temperature was maintained throughout the experiment. The moment of the temperature shift to 37 °C is referred to as 0 min time point. DiO-SFV was detected with a FITC filter, while DiO-SFV-FND was detected with FITC and A594 filters, separately. An image series of the fluorescent emission was recorded with a charge-coupled-device camera at 1 frame per 5s for a total of 25 min. Before and after fluorescence imaging, the localization of the nucleus and plasma membrane of the cell was determined by differential interference contrast (DIC) imaging. As shown in Fig. S2 and Fig. S3, the cell shape and position did not change significantly during the fluorescence recording period.

As a negative control, BHK-21 cells were treated with 20 mM ammonium chloride (Sigma) before incubation with virus or virus-diamond conjugates [30,31]. To make sure that the long duration imaging does not bleach the DiO-SFV fluorescence, imaging was also performed on DiO-SFV and DiO-SFV-FND alone. The data produced during the experiments consist of a time series of ∼300 images that show several fluorescent particles in the same imaging field. To further analyze the change in fluorescence intensity as a function of time, first, we manually found the coordinates of the possible fusion events where fluorescence intensity was observed to increase. Next, a region of interest (ROI) was formed near these coordinates to calculate the intensity value using Fiji (fiji.sc/).

### 2.8 Determining the relative location of DiO-SFV-FND

To obtain the position information of the conjugates, especially the location of FND where we carried out our T_1_ experiments on SFV-FND, BHK-21 cells were incubated with DiO-SFV-FND. After co-culturing for different times (0, 2, 4, and 6 hpi), the cells were fixed with 3.7% paraformaldehyde solution for 15 min, followed by staining the endosomes using EEA1 monoclonal antibody (4 μg/mL, ThermoFisher), and goat anti-Rabbit IgG (H + L) cross-adsorbed secondary antibody, Alexa Fluor 350 (5 μg/mL, ThermoFisher), respectively. 0 hpi samples were obtained by attaching DiO-SFV-FND on BHK-21 cells on an ice bath for 45 min, immediately followed by the same fixing and staining protocol. The cells were then imaged using a fluorescent microscope (DeltaVision Elite). Signals from DiO, FND, and stained endosomes were obtained with FITC, A594, and DAPI filters. Images were analyzed using FIJI to determine the relative position for conjugates and endosomes.

## 3 Results and discussion

### 3.1 Using relaxometry and SFV – BHK-21 as a model system

SFV is a positive-stranded RNA virus of the genus Alphavirus of the *Togaviridae* family and one of the least pathogenic alphaviruses for humans [32]. Its popularity stems from its broad host range and similarities to other pathogenic viruses such as chikungunya virus and O'Nyong-Nyong virus. Moreover, SFV grows to high titer in cultured cells and one does not require high containment laboratory facilities to work with it, which makes it a very practical virus [24]. Likewise, BHK-21 is a widely used cell line in virology research and it ingests FNDs without any additional procedure. Moreover, BHK-21 cells have also been shown to be very permissive to SFV by Helenius et al. and they are commonly used to propagate and to study this virus. It has been shown that the entry of SFV in BHK-21 occurs within an hour after infection and infection proceeds very rapidly as virus progeny can be observed in the medium within 3 h post infection [33]. Hence, BHK-21 and SFV is a perfect model system for this work.

Next, we briefly explain the relevance of relaxometry in free radical sensing. A schematic representation of probing the general intracellular free radical response (with bare FNDs) and free radical response near viral particles (with FND-SFV conjugates) is shown in Fig. 1. The core principle of this method is that the transition between polarized and equilibrium state changes based on the spin noise from radicals in its surrounding. The electron energy level diagram and the detailed photophysics of the NV center is further explained in supplementary information (S) in **Section S4**.

### 3.2 SFV-FND conjugates

**Fig. 2:**
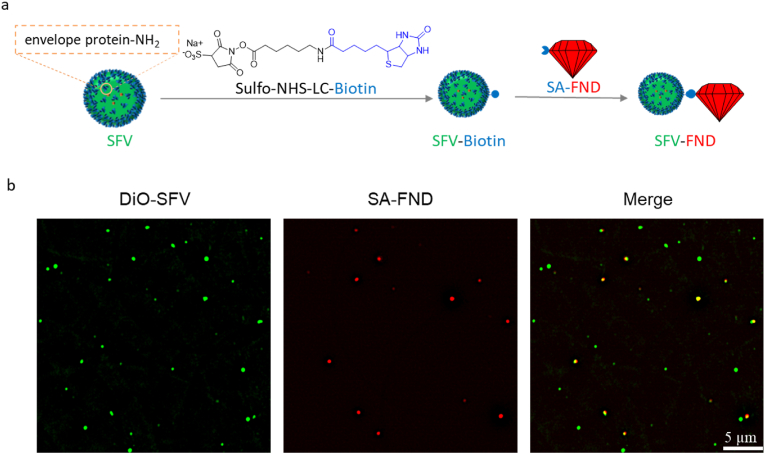
(a) Schematic representation of SFV-FND formation. (b) Represent fluorescent images of SFV-FND conjugates.

The biotin-streptavidin interaction is widely used in biological experiments, especially in the biohybrid materials field [[25], [26], [27],^34^]. Here, we used the interaction between biotin and streptavidin to conjugate SFV and FND (Fig. 2a). As shown in Fig. 2b, Biotin-SFV was labeled with DiO (green color), and SA-FND yielded red color. The yellow dots indicate the merged signal of DiO-SFV and SA-FND, which represents the colocalization of SFV and FND. The viral particles are in 10-fold excess over FND particles to guarantee that the following T_1_ experiment was done on an SFV-FND conjugates. The number of viral and diamond particles was calculated using the methods as described by Hilde et al. [31] and Barton et al. [35]. It can be clearly seen that all the FND particles are colocalized well with SFV, while some free viral particles can be observed individually.

### 3.3 Kinetics of virus entry into the cell, infection duration and determining the degree of oxidative stress caused by SFV infection

**Fig. 3:**
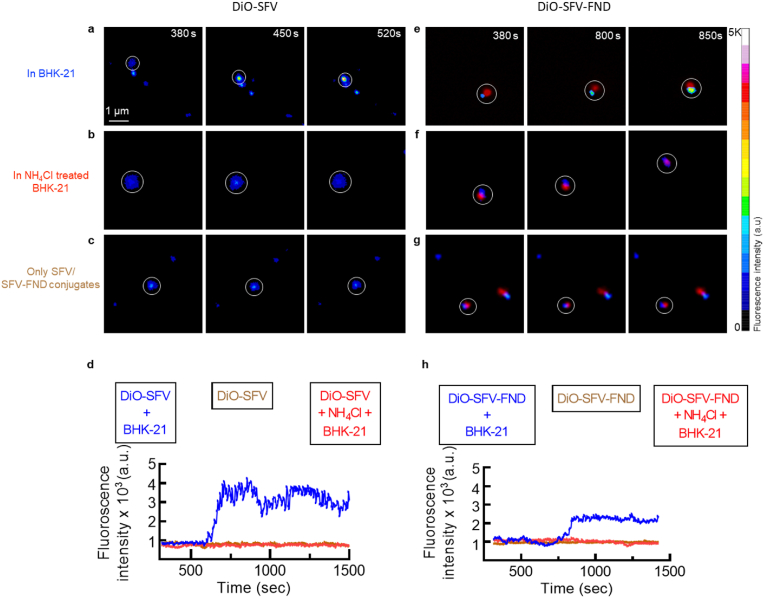
The time required for SFV and SFV-FND to enter the cell. Representative time-lapse image sequences demonstrating the change in intensity of the DiO fluorescence after (a–c) adding the DiO-SFV or (e–g) DiO-SFV-FND to the cells. In (b) and (f) we show the time series when the uptake is inhibited with NH_4_Cl. (c) and (g) show the respective particles in an empty Petri dish (control). Change in normalized fluorescence intensity with respect to time. (d) and (h) show the data for DiO-SFV and DiO-SFV-FND respectively. (a complete time series is shown in the supplementary material in **Section S2**).

After determining the titer of the virus stock solution to be ∼2 × 10^9^ TCID_50_/mL, we measured how long it takes for a virus particle to enter the BHK-21 cells. Here we used a method previously reported by Hoornweg et al. [36] In particular, we monitored the fluorescence intensity of the DiO-label attached to the sucrose purified SFV that was added over the BHK-21 cells. A time-lapse image sequence in Fig. 3a shows the sudden burst in fluorescence around ∼600 s into the experiment. This indicates the virus membrane fusion as the fluorescence intensity increases due to the dilution of the probe in the endosomal membrane. The self-quenching property of DiO dye was proven in Fig. S1c. Fig. 3b shows the fluorescence intensity of DiO-labeled virus when cells were treated with NH_4_Cl, which inhibits the virus fusion [37]. This is a negative control used to confirm the association between the fluorescence burst and the virus fusion.

In addition, the entrance of virus-diamond conjugates was also performed to check whether the conjugation will affect the virus internalisation. Fig. 3e shows the sudden increase of the DiO intensity at around 800 s. This is around 200 s delay compared with DiO-SFV group. According to Kennie's report that the mean endocytosis and fusion time in SFV-BHK-21 cells model is 10 min and 4 min [38], respectively. The fusion of conjugates happens around 800 s, which means the total time for endocytosis and fusion, and this event is in the range of previous reports. While Fig. 3f shows that NH_4_Cl also inhibits the fusion of SFV-FND. In conclusion, the characteristic timing of early infection of the virus is similar for conjugates. There is around 4 times fluorescence increase for DiO-SFV's fusion, while there is only 2 times increase for the DiO-SFV-FND group (Fig. 3d, h). One possible reason is that viral particles were modified with biotin before DiO staining. This step may decrease the staining effect. Additionally, the attached particle reduces the space that is available for the label as well. Furthermore, it was confirmed that optical bleaching of the DiO does not occur under the experimental conditions we used (Fig. 3c and g).

### 3.4 Measuring free radical response of BHK-21 cells to SFV infection at discrete timepoints at random locations and its effect of SFV infection on cytoplasmic transport

Next, we investigated the general free radical response of BHK-21 cells during SFV infection at a random location. Here, we infected the cells which already contained intracellular FNDs with SFV with a MOI of 20. MTT and morphology assays (Figs. S11a–d) showed that this infectious condition has no influence on cell viability before 10 h post infections. We did not observe any significant changes in radical loads in random locations of untreated cells (negative control), cells incubated with UV-inactivated virus particles, and cells challenged with infectious virus particles.

**Fig. 4:**
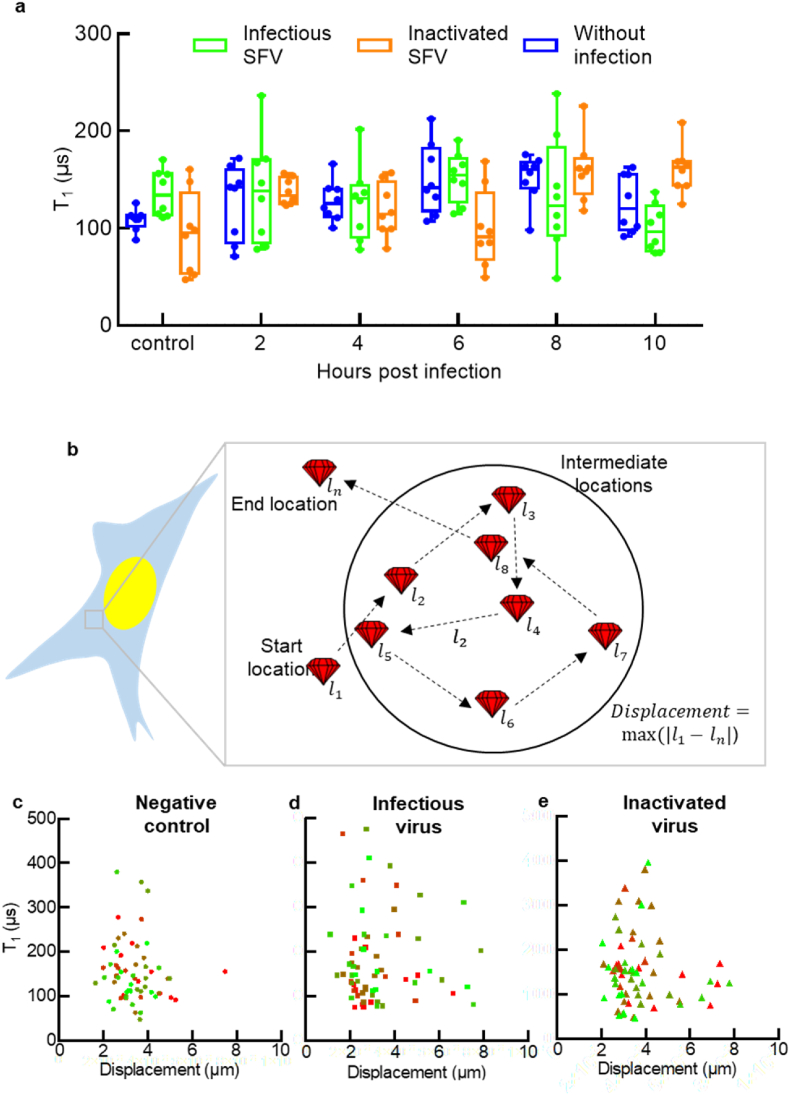
Individual T_1_ measurements recorded every 2 h post infection (hpi) till 10 hpi in untreated cells (negative control), incubated with inactivated SFV and challenged with infectious SFV. All the timepoints across all the groups have at least 8 independent measurements. (b) Graphical representation of the FND movement within the cell cytoplasm to evaluate whether more active areas of greater movements were experiencing different T_1_ times. We plot T_1_ relaxation correlated with the maximum FND displacement for the cells treated with (d) plain cell-culture medium (negative control), (e) infectious SFV and (f) inactivated SFV group. Green and red color represent 0 hpi (time control) and 10 hpi, respectively. The off-green and red colors indicate the intermediate timepoints. Plots in b-d, have ∼40 data points each obtained in three independent experiments. (For interpretation of the references to color in this figure legend, the reader is referred to the Web version of this article.)

During relaxometry measurements (Fig. 4a) we did not observe any significant differences between radical loads at random locations.

While applying the pulsing sequences on an intracellular FND for measuring the relaxation time (T_1_), we continuously track the movement of the FND within the cell cytoplasm. Specifically, after every 3 to 5 s, we scan the fluorescence intensity within the 2 × 2 × 1 μm volume around the last known position of the particle. Subsequently, the new position of the FND is determined in two steps- (i) using a gaussian approximation to determine the brightest voxel in X and Y direction (ii) finding the brightest voxel at different Z coordinates for the X and Y coordinates obtained in the previous step. Tracking FND's movement within the cytoplasm allows us to further investigate the effect of viral infection on cytoplasmic transport. Diffusion of viral genetic material within the cytoplasm has been an important topic of experimental or computational investigation [[39], [40], [41], [42]] as viral genetic material needs to be transported from outside of the cell to the nucleus in order to initiate the viral activity. On the other hand, diffusion of small molecules within the cytoplasm using electron spin resonance [43] or fluorescence-based imaging methods [44,45] has also been a topic of great interest. Most of the experimental work in these areas utilizes fluorescent labels, which are prone to bleaching. Moreover, these studies focus on movement of a single small molecule in the cytoplasm and no studies explore the systemic alterations in the cytoplasmic transport caused by the external stimulus (viral infection in this case).

Cytoplasmic transport can be assessed by a variety of ways such as quantifying the particle velocity, diffusion rate or displacement. Here, we quantify the maximum diamond displacement within the cell as shown in Fig. 4b. First, we manually identified the particle, hence it's coordinates at the starting position l_1_, within the cell at the beginning of the experiment. Then we optically followed the particle to track its coordinates li while the T_1_ measurements were recorded. Then we calculated the maximum distance traveled by the particle with respect to the initial position. The plots in Fig. 4c–e depict T_1_ relaxation against the maximum displacement determined for every individual FND for all measured timepoints. The idea behind this was to see whether areas with increased activity (indicated by displacement) would differ in T_1_. From the results it can be clearly seen that all the points form a dense cluster for the negative control group where T_1_ and the maximum displacement varies between 100 and 200 μs and 2–4 μm respectively. On the other hand, two components influencing the maximum displacement are (i) physical movement of an FND and (ii) an uncertainty in optical refocusing deployed for diamond tracking. We recorded a maximum displacement of ∼ 1–2 μm even for a stationary particle placed in a dry Petri dish. A similar cluster was observed for the inactivated virus and infectious virus group. Interestingly, the data points in those two groups were found to be more scattered compared to the negative control group indicating that FND movement in the cytoplasm is elevated upon introduction of the external stimulus. In Fig. 4c–d, green and red color represent the control and 10 hpi timepoint respectively whereas color shades in between green and red indicate the intermediate timepoints. From Fig. 4c–d it is clear that the T_1_ x displacement plane is not divided in “distinct colors” which means that no correlation between FND movement and the hpi can be drawn.

This method can easily be extended to both quantitatively and qualitatively investigate the cytoplasmic transport. Due to FND's photostable fluorescence, the same FND can be tracked continuously before and up to ∼24 h (based on the work in our group) after adding the virus. Uncertainty in optical refocusing can also be minimized by suitably modifying the tracking algorithm. Such measurements can potentially reveal the diffusion rate and particle velocities at high time resolution.

### 3.5 Position of SFV-FND conjugates

**Fig. 5:**
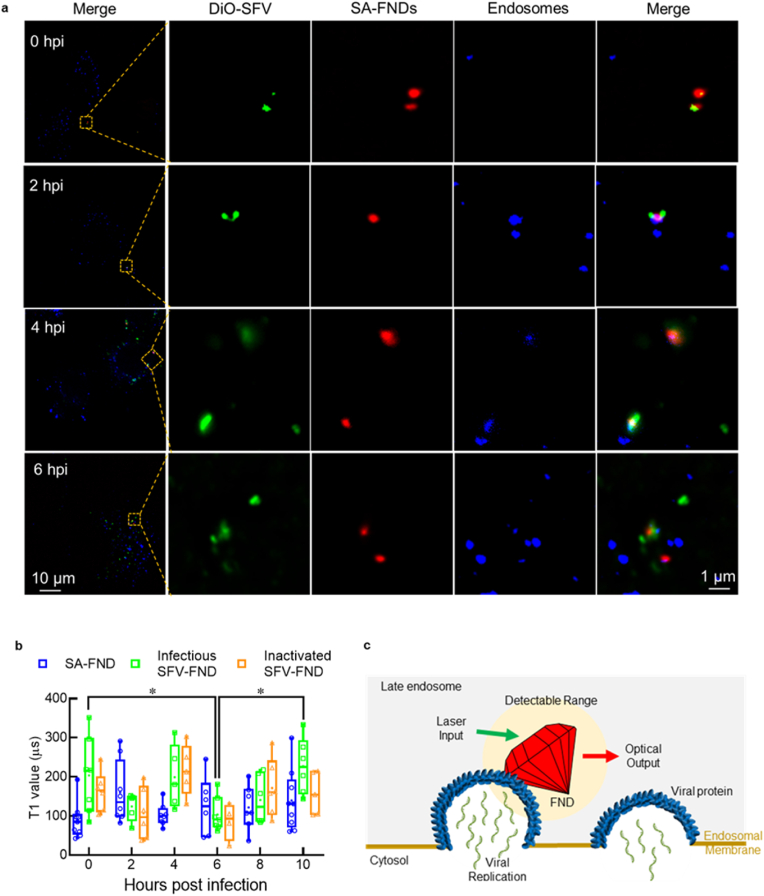
(a) Representative fluorescent images of SFV-FND conjugates in BHK-21 cells at different incubation times. 0 hpi represents DiO-SFV-FND conjugates and BHK-21 cells were co-cultured at 4 °C for 1 h; 2, 4, and 6 hpi represent DiO-SFV-FND conjugates and BHK-21 cells co-cultured for 2, 4, and 6 h at 37 °C, 5% CO_2_, followed by fixing and staining. (b) T_1_ measurements recorded from 2 hpi to 10 hpi during incubation with infectious SFV-FND and inactivated SFV-FND. Cells treated with SA-FND were set as negative control group. All the timepoints across all the groups have at least 8 independent measurements. (c) Representation of the location and events that diamond particles are detecting by using SFV-FND. * indicates statistical significance as determined by the one-way Anova test. A two-way Anova with post-hoc test (Sidak) indicates that time has a significant effect on T_1_ but infectious and inactivated viruses cause similar responses (P = 0.0229, *).

In the previous section we investigated free radical generation at a random location which is not necessarily close to the point of virus entry. Here, virus-diamond conjugates were formed to investigate the intracellular free radical response near viral particles, as diamond can detect the magnetic noise within 10 nm range. To clarify the position of SFV-FND conjugates and the location where we carried out our T_1_ experiments, BHK-21 cells were incubated with DiO-SFV-FND for different times, followed by a fixing and staining process. For the control group, the uptake of DiO-SFV-FND conjugates was blocked when the temperature was lowered to and kept at 4 °C for 1 h. As shown in Fig. 5a (0 hpi), the conjugates (red and green dots) are not colocalized with the endosomes (blue dots), and the location of conjugates is closer to the cell membrane. This is consistent with previous reporting the timing of virus entrance [38]. The conjugates can be found in the endosomes at 2, 4, and 6 h post infection. As shown in Fig. 5a (2, 4, and 6 hpi), FND, endosomes, and part of SFV colocalized well. Also, part of the green signals appears out of the endosome. This could be some DiO dye interacting with other intracellular lipids after membrane fusion occurred. FNDs are always colocalized well with endosomes. It is worth noting that replication of alphaviruses, including SFV, takes place in cytoplasmic viral factories at the outer surface of endosomes after membrane fusion [46]. To conclude, we can investigate the free radical response of BHK-21 cells during gene replication by using SFV-FND, as demonstrated in Fig. 5c.

### 3.6 Measuring the free radical response of BHK-21 cells to SFV-FND infection at discrete timepoints near viral particles

We investigated the intracellular free radical response near viral particles using SFV-FND conjugates at discrete time points post SFV infection with MOI 20. Comparing with Section 3.4 where measurements were conducted on bare FNDs at random intracellular locations, measurements on SFV-FND reveal the radical response near viral particles. As a result, T_1_ values detected using SFV-FND are more affected by the infectious events. In Fig. 5b we observe a lower T_1_ value at 2 hpi (145 μs), compared with 0 hpi (220 μs). At 2 hpi is the time when SFV is completely uncoating. The latent period during the growth of SFV was 3 h. However, the traditional method based on RNA or protein techniques cannot detect the potential crisis, as viral replication and other activities can evade detection of immune responses from host cells, while relaxometry is sensitive to relatively early processes of radical formation. Fig. 5b also shows the lowest T_1_ values (highest radical load) at 6 hpi (91 μs). This is also the timing when the viral load is greatest as described by Kaariainen et al. [47]. According to Akaike's work [7], viral replication directly leads to the production of iNOS (inducible NO* synthase), resulting in NO* (nitric oxide) overproduction. NO* is a gaseous nitrogen-centered radical that is formed during viral infections. This radical is also a source of cellular oxidative stress (as described in Fig. S4C). Our findings support the hypothesis that RNA viruses utilize oxidative stress induced during infection to help temporally control genome RNA capping and genome replication [48].

From 6 hpi to 10 hpi the radical load is decreasing. This decrease might be due to a decrease in virus-producing rate or cells gradually losing their ability to produce radicals at a high viral load. Also the radicals produced in 6 hpi might be gradually transformed into non-paramagnetic ROS. This is also supported by the DCFDA assay (to measure both paramagnetic and non-paramagnetic ROS) in **Section S7.** As shown in Figs. S7b–c, DCFDA treated infectious BHK-21 cells at 10 hpi are brighter than at 5 hpi.

UV irradiation can damage the genome of the virus, which will inhibit viral replication. However, not only the viral replication but the viral components can also induce NO* overproduction. As a result the cell's response to UV-inactivated SFV-FND conjugates is similar to infectious SFV-FND. A similar response is seen also in inactivated viral vaccines where cells also respond without the actual infection. As a comparison, the control group (BHK-21 cells with SA-FND) did not show any significant changes (Fig. S5). The same is true for bare FNDs which are not measuring at the location of virus entry. This indicates that the radical response to viruses is localized rather than throughout the entire cells.

### 3.7 Investigating pseudo real-time variation in intracellular free radical amount post SFV infection

**Fig. 6:**
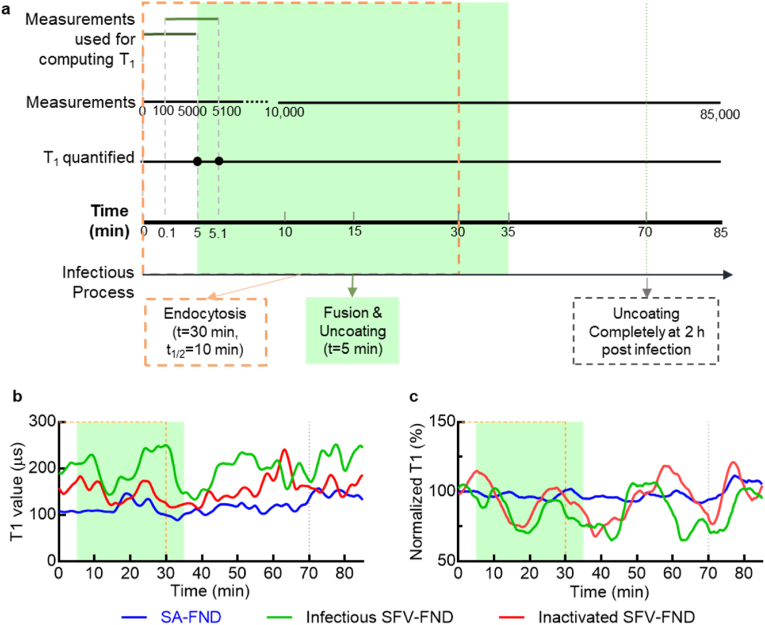
(a) Graphical representation of the ‘moving window’ algorithm used for analyzing the data to deduce pseudo real-time variation in the intracellular free radical concentration near viral particles. Time-dependent early viral infection process was superimposed to ‘moving window’ algorithm. The orange dash square represents the endocytosis process, and green square represents the fusion & uncoating process, respectively. (b) Experimental results obtained for cells treated with infectious SFV-FND, inactivated SFV-FND, and SA-FND (negative control). The data was shown as median of at least 8 independent measurements for each group. (c) Average of independent normalized T_1_ curves for each group. Two-way Anova with post-hoc test (Turkey) indicates the significant difference between infectious/inactivated SFV-FND and SA-FND groups (P < 0.001, *). (For interpretation of the references to color in this figure legend, the reader is referred to the Web version of this article.)

After probing the intracellular free radical concentration in BHK-21 cells every 2 h post SFV infection and the corresponding FND movement, we evaluated the “pseudo real-time” free radical response of host cells to the intruding virus. In these experiments, we apply the T_1_ measurement cycle for 85,000 times in 85 min. Then we deployed the “moving window algorithm” to analyze the collected data to investigate the pseudo real-time evolution of the T_1_ relaxation which is explained in Fig. 6a. Instead of using output of all of the T_1_ measurement cycles for quantifying the T_1_ value, a small subset was used for quantifying the T_1_ to show the rolling average. Therefore, this analytical method does not necessarily improve the temporal resolution in a conventional sense, but it does provide the evolution of T_1_ as a function of time. Figs. 4a and 5b.

SFV-FND conjugates were used to investigate the radical response at the place of virus entry. The variation in T_1_ obtained from BHK-21 cells treated with infectious SFV-FND (green, Fig. 6b–c) is significantly different from SA-FND treated cells (blue, Fig. 6b–c). We observed a decrease of T_1_ between 10 and 15 min (from 248 to 133 μs) and during 30–40 min (from 270 to 130 μs) when cells were treated with infectious SFV-FND. This decrease did not occur in the control group. Interestingly, these changes in radical load are occurring during the fusion and uncoating period in the early SFV infectious process (see pseudo real-time scale Fig. 6a). Normally, it takes around 30 min to internalize all the prebound viral particles and half of the viral particles are ingested at around 10 min [38,49]. Membrane fusion and uncoating (with a mean time of 5 min) takes place immediately after endocytosis. According to Mark et al. [49], most fusion events of SFV take place during ∼7–25 min, which indicates a higher possibility of observing a membrane fusion event during this period. The dropping period (10–15 min) is also consistent with the fusion time (around 800 s) for SFV-FND which we investigated in the entrance experiment described in Section 3.3 **(**Fig. 3**e).** Moreover, stress granules (SG) formation occurs transiently after viral uncoating but before viral mRNA transcription to shut the host protein synthesis off [50,51]. One inducer of SG formation is oxidative stress [52]. As a result, the decreasing T_1_ at 10–15 min can probably be attributed to the fusion event, while the decreasing T_1_ at 30–40 min is probably due to the SG formation after fusion and uncoating. The variations were also shown in inactivated SFV-FND treated cells, but with less amplitude comparing the red and green curve in Fig. 6b.

Viral membrane fusion and uncoating are interesting processes in the early-phase of infections. Widely-used methods to detect this processes are electron microscopy [53,54] and fluorescent microscopy [31,36] based on self-quenching dyes (as shown in Fig. 3). From our results we hypothesize a relation between radical response and viral membrane fusion and uncoating.

### 3.8 Influence of pH, temperature, viscosity and other factors on T_1_ relaxation

Last year, Fujisaku and co-workers demonstrated how the T_1_ relaxation of 50–100 nm FND is affected by pH. In fact, the authors proposed a diamond magnetometry based pH-metry to measure pH changes in the range of 3–11. On the other hand, virus infection has been shown to alter the intracellular [55,56] and extracellular [57] pH by up to a unit (from pH of ∼7.4). Although, the necessity of low pH for SFV fusion is discussed in the literature [33,58], whether SFV infection actively modifies the intracellular pH is not yet clear. However, from our previous measurements of pH versus T_1_ we conclude that T_1_ was not influenced by pH significantly in the relevant pH range [59].

Similar to pH, temperature also affects the coherence time of the NV centers. The application of NV-based thermometer for the intracellular temperature sensing was also demonstrated [60]. In addition, molecular interaction, solvent, or solvent viscosity affect the NV coherence as well [61]. However, all these factors are unlikely to change drastically enough to cause a shift in T_1_ inside a cell due to the viral infection. Hence, their effect on T_1_ observed during our measurements can be disregarded. Furthermore, it has been shown that ROS is often involved in the signaling pathway while cells cope with the photo illumination [62,63]. Therefore, cellular phototoxicity might also be a factor to be considered if pseudo real-time T_1_ data is to be acquired over longer durations. In fact, as certain cell types are more susceptible to phototoxicity than others [64], cellular phototoxicity might still be a relevant factor even for a short T_1_ measurements depending on the experimental design (cell type, laser power, etc). However, artifacts generated due to photo-induced free radical formation were excluded by including a control sample (where only time was passed and no interventions were made).

## 4 Conclusions

Here we have demonstrated the utility of relaxometry to study unprecedented details of the free radical response of host cells upon viral infection. Using SFV and BHK-21 as a model system, we probed the free radical response of the cells until 10 hpi. The general intracellular radical response was investigated by treating host cells with FND and SFV separately, and intracellular radical response near viral particles was investigated by virus-diamond conjugates (SFV-FND).

(i)When BHK-21 cells were treated with FND and SFV separately, we found that the free radical concentration in infected host cells does not change significantly. Moreover, we reveal the unique potential of this method to probe the systemic alterations in the cytoplasmic transport. SFV infection was found to elevate the FND movement within the cytoplasm as quantified in terms of maximum particle displacement. (ii) By using SFV-FND, relaxometry is able to monitor the radical variation at the site of viral production during viral replication and even in the early infectious process. We observed alterations of T_1_, which represent the intracellular free radical concentration near viral particles. This is not possible with any other method. Immunostaining was used to determine where we conduct the T_1_ measurements. Moreover, a pseudo real-time technique based on relaxometry and virus-diamond conjugates was used to detect radical formation during viral membrane fusion and uncoating events from cellular radical response. Obtaining such information with the conventional techniques is impossible.

Going forward, efforts need to be taken to (1) efficiently track the movement of the FND (2) implement sophisticated pulsing techniques such as double electron-electron resonance measurements for identifying specific free radical species and enhancing specificity and (3) reduce the FND-to-FND variation in its quantum sensing properties. Finally, we hope that this novel and powerful method will enable researchers to discover and/or redefine the fundamentals of the viral infection and thereby contribute to the design of strategies to combat them.

## Declaration of interests

The authors declare that they have no known competing financial interests or personal relationships that could have appeared to influence the work reported in this paper.
