# Supplementary material for: Applying NV center-based quantum sensing to study intracellular free radical response upon viral infections
Source: Redox Biol. 2022 Mar 18;52:102279. doi: 10.1016/j.redox.2022.102279 (PMC8965164; doi:10.1016/j.redox.2022.102279)
Supplement: Multimedia component 1 [file mmc1.docx]

Supporting Information

Applying NV center-based quantum sensing to study intracellular free radical response upon viral infections

Kaiqi Wu, Thea A. Vedelaar, Viraj G. Damle, Aryan Morita, Julie Mougnaud, Claudia Reyes San Martin, Yue Zhang, Denise P.I van der Pol, Heidi Ende-Metselaar, Izabella Rodenhuis- Zybert and Romana Schirhagl*

**S1. Characterization of the DiO-labeled SFV**


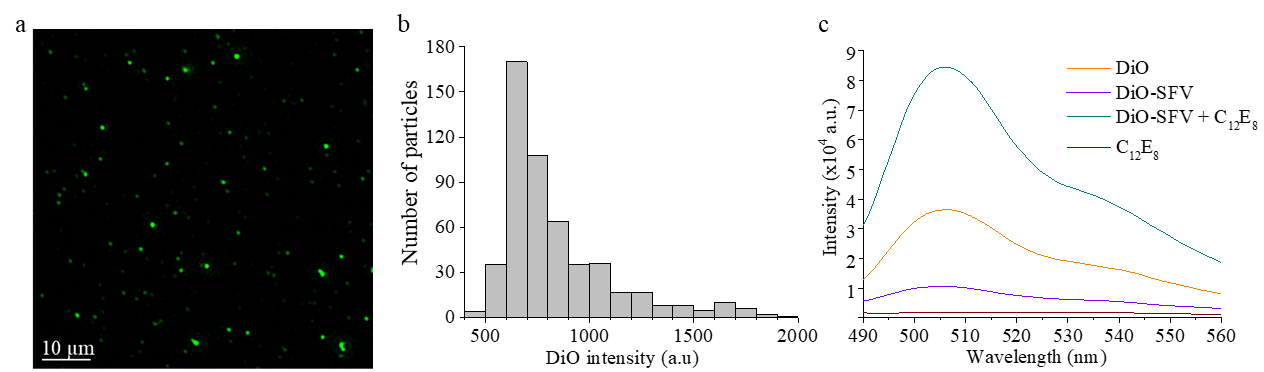


**Figure S1**. Characterization of DiO-labeled SFV. (a) Representative images showing DiO-labeled SFV particles. (b) The intensity of DiO-labeled SFV was determined with fluorescence microscopy. Particles with low fluorescence intensity (<1000 A.U.) were selected for viral fusion experiments and subsequent image analysis. (c) Fluorescence emission spectra of DiO-labeled SFV. DiO-labeled SFV was mixed with/without C_12_E_8_ in HNE buffer. Samples only containing C_12_E_8_ or DiO were set as negative or positive control groups, respectively. Emission scans were recorded at wavelengths 490 to 560 nm with excitation at 480 nm.

DiO, a self-quenched dye, was used to investigate the early infection process, including the membrane fusion properties. First, SFV were labeled with a relatively high surface density of the fluorescent probe DiO in the viral membrane. This way, its fluorescence was largely quenched but still allowed single virus particles to be clearly detected (**Figure** **S1a, b**). Membrane fusion of virus particles labeled were observed as fluorescence dequenching due to the dilution of the DiO probe into the target membrane. A significant increase in DiO fluorescent intensity was observed when the viral membrane was dissolved by the addition of C_12_E_8_ detergent (a nonionic detergent) with respect to the dequenching of DiO (**Figure** **S1c**).

**S2. Increase in fluorescence intensity of the DiO upon viral fusion as observed with fluorescence microscope**


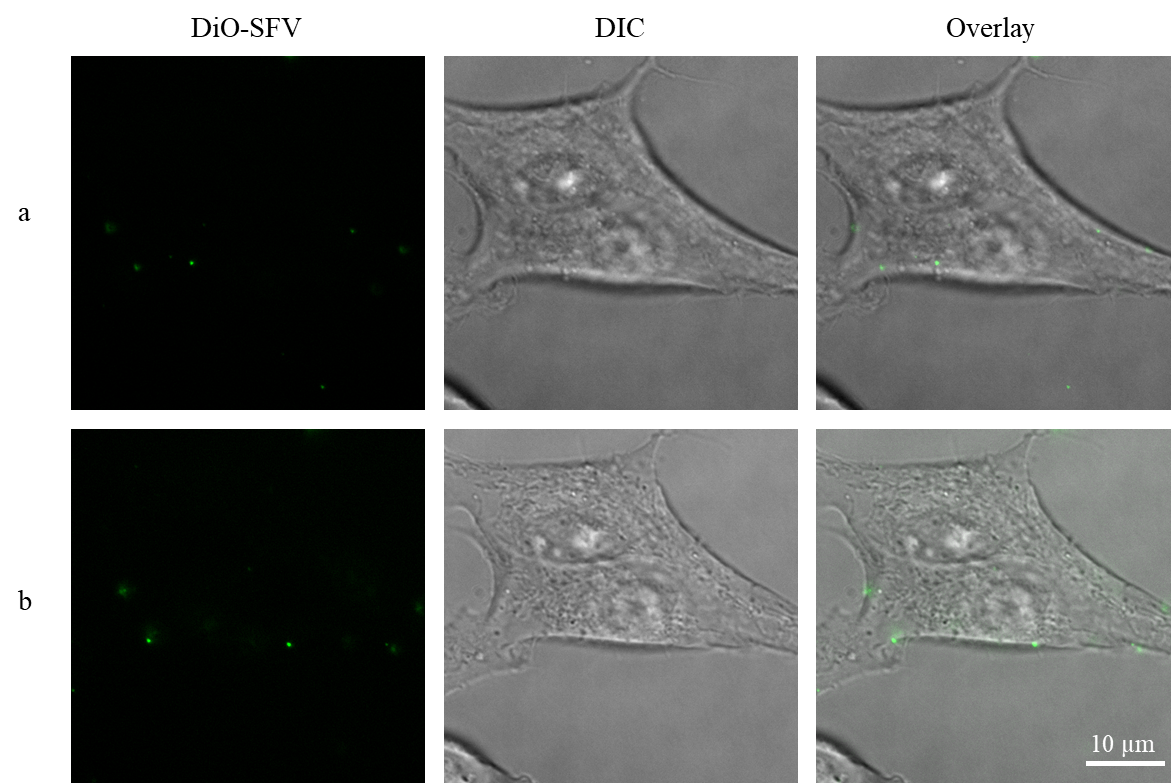


**Figure S2**. Viral fusion assay of DiO-SFV. Snapshots of BHK-21 cells recorded (a) after 45 minutes infection at 4°C and (b) after another 30 minutes infection by DiO-SFV at 37°C. Increase in fluorescence intensity is clearly visible after 30 minutes of incubation at the elevated temperature. The DiO signal was recorded in the FITC channel of fluorescent microscope (DeltaVision Elite). Cellular morphology was also recoded before and after viral fusion to access cellular health. The scale bar indicates 10 µm.


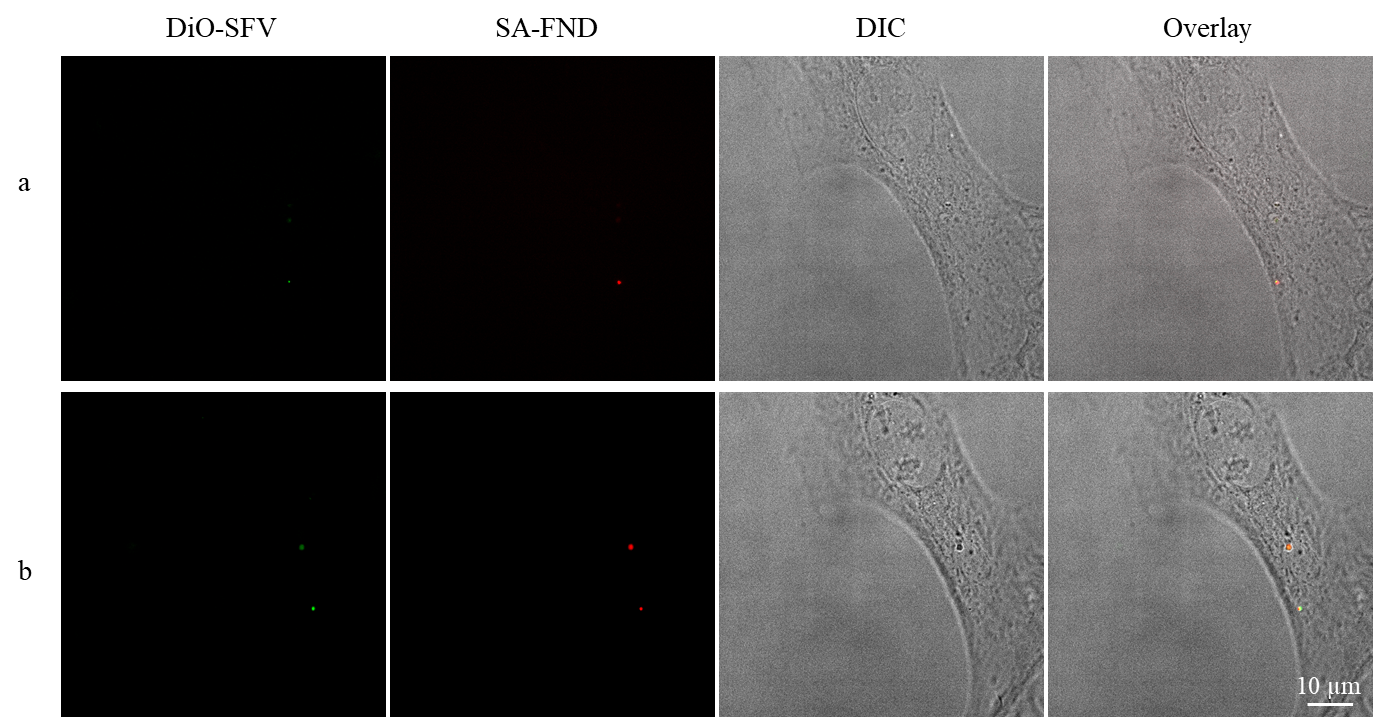


**Figure S3**. Viral fusion assay of DiO-SFV-FND. Snapshots of BHK-21 cells recorded (a) after 45 minutes at 4°C and (b) after another 30 minutes of infection by DiO-SFV-FND at 37°C. An increase in fluorescence intensity is clearly visible after 30 minutes of incubation at the elevated temperature. Signals for DiO and SA-FND were recorded in the FITC and A594 channels of a fluorescent microscope (DeltaVision Elite). Cellular morphology was also recoded before and after viral fusion to access cellular health. The scale bar indicates 10 µm.

Videos are available online which highlight the variation in DiO fluorescence intensity when cells were treated with DiO-SFV/DiO-SFV-FND, first with NH_4_Cl followed by DiO-SFV/DiO-SFV-FND, and DiO-SFV/DiO-SFV-FND without cells.

**S3. Summarized process of BHK-21 infected by SFV and mechanisms of cellular oxidative stress in virus infection**

The SFV-BHK-21 infectious system is a well-studied model in virology. The whole infection process includes both a latent period and a viral replication process. The latent period consists of viral particles binding on the cell membrane, entering into cells by endocytosis, membrane fusion and uncoating, and releasing of genetic materials into the cytoplasm. Within 2 h post infections viruses are completely uncoated. Virus concentrations increase exponentially from 3.5-4 h post infections, and the greatest yields were reached between 6 and 7 h post infections (**Figure S4a, b**). **Figure** **S3c** shows how virus infection induces cellular oxidative stress. Specifically, viral replication and other viral components directly lead to the production of iNOS (inducible NO* synthase), resulting in NO* overproduction, which is the source of infected cellular oxidative stress[1-2].


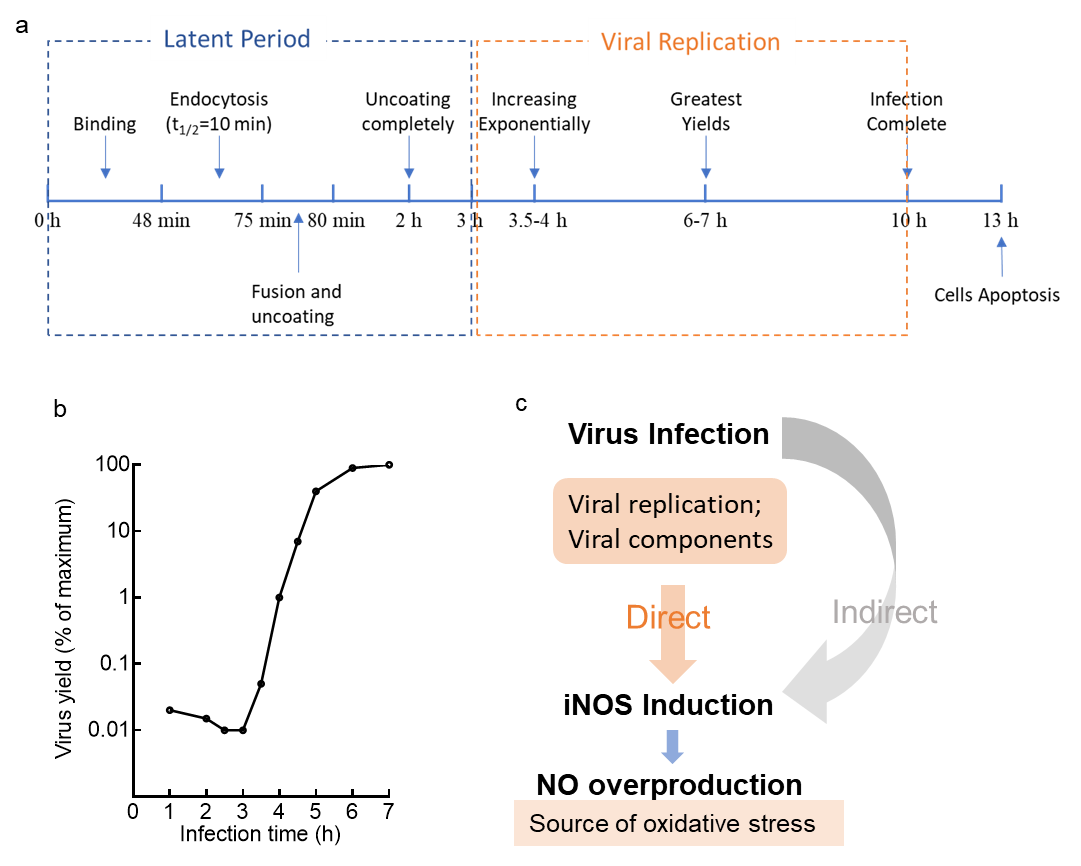


**Figure S4**. (a) Summarized timescales for BHK-21 infection by SFV (according to[3-5]). (b) Growth curve of SFV in BHK-21 cells infected at a MOI=20 [4]. (c) Mechanisms of cellular oxidative stress in virus infection[1-2].

**S4. Principle of relaxometry relevant to the intracellular free radical sensing upon viral infection**


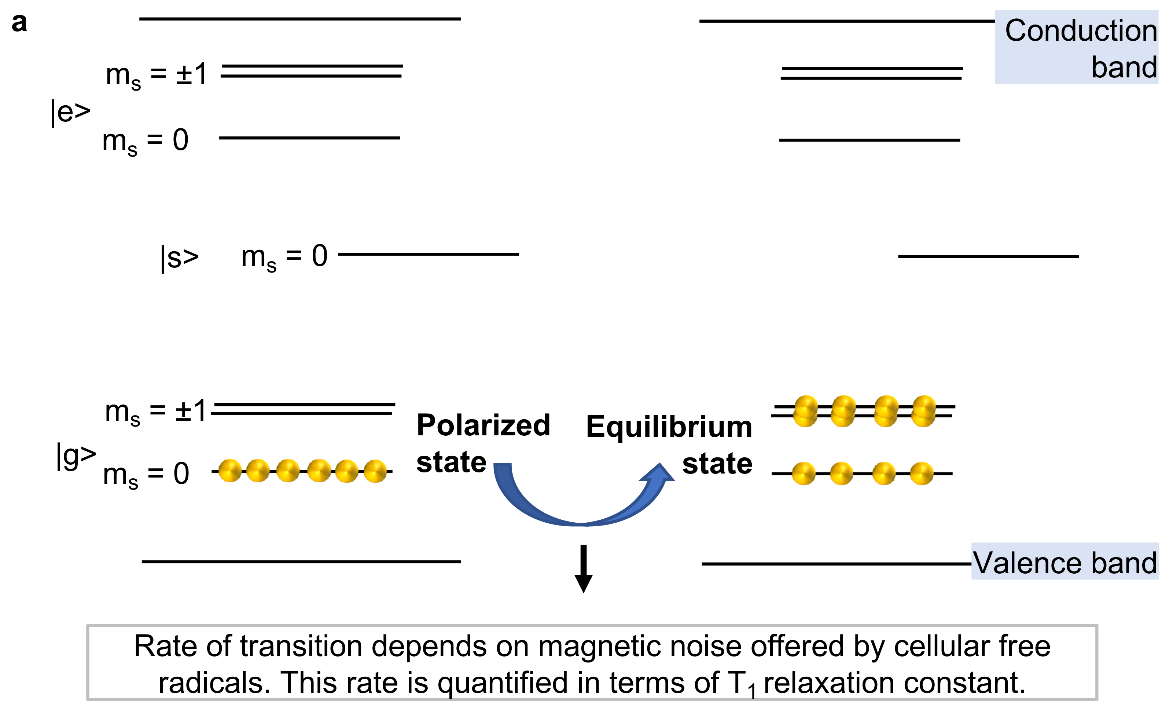


**Figure S5.** Photophysics of relaxometry.

Basic photophysics of relaxometry that is relevant for this work is explained using **Figure** **S5**. NV defects embedded in FNDs can be pumped into the optically brightest polarized state under a continuous laser illumination where most of the electrons are populated in the |g m_s_ = 0 energy state. Once the laser is turned off, the optically brightest polarized state of the NV center is lost and electrons transition back to their original equilibrium states |g m_s_ = 0 and m_s_ = ±1. The time required for the transition from the polarized state to the initial unpolarized equilibrium state is dependent on the magnetic noise around the NV defect. As unpaired free electrons of intracellular free radicals offer magnetic noise, they alter this transition time. In particular, the higher the free radical concentration around the NV center, the higher is the magnetic noise hence shorter is the transition time and vice versa. As a result, the transition time is a direct measure for the amount of cellular free radicals in the vicinity of the NV center. We quantify the transition time between polarized and equilibrium state via measuring spin-lattice or T_1_ relaxation.

**S6. Fluorescence assay-based evaluation of SFV infection induced oxidative stress in BHK-21 cells**

S7.1. Experimental method

Oxidative stress in BHK-21 cells at different timepoints (0, 5, and 10 hpi) in the SFV infection cycle was probed using the DCFDA fluorescence assay (Thermofisher, Netherlands). The experimental groups used in this experiment at different timepoints are shown in **Figure S6**a and **Figure S6b** indicates the timeline of the experiment. In short, 40,000 BHK-21 cells were plated in 35 mm glass bottom petri dishes (Greiner bio-one, Germany) one day before the experiment. Approximately seven hours post seeding, we incubated the cells with 1 μg/ml 70 nm FND suspension. After overnight incubation, the FND suspension was replaced either with 500 µl fresh medium or with 100 µl of 2% infection medium. For the infection groups, 10% RPMI medium was added over the cells one hour post infection. At different timepoints, cells were first washed with the washing solution (2% FBS in PBS) three times and then incubated with 100 µl of 10 µg/mL DCFDA for one hour. The positive control group was subsequently treated with 0.01% H_2_O_2_ (in washing solution). The positive control group was treated with DCFDA one hour prior to the rest of the groups so that all the groups can be imaged at the same time. We note that, cells were maintained in the incubator at all times. At the end, cells were washed and imaged using a laser scanning confocal microscope (Zeiss LSM780, Germany) at the excitation wavelength of 488 nm. The same image acquisition settings (Gain 700 and laser power 2.1) were applied while imaging different groups at different timepoints. At every timepoint, at least four images of every group were recorded. This entire experiment was repeated two independent times to collect eight images per group per timepoint.


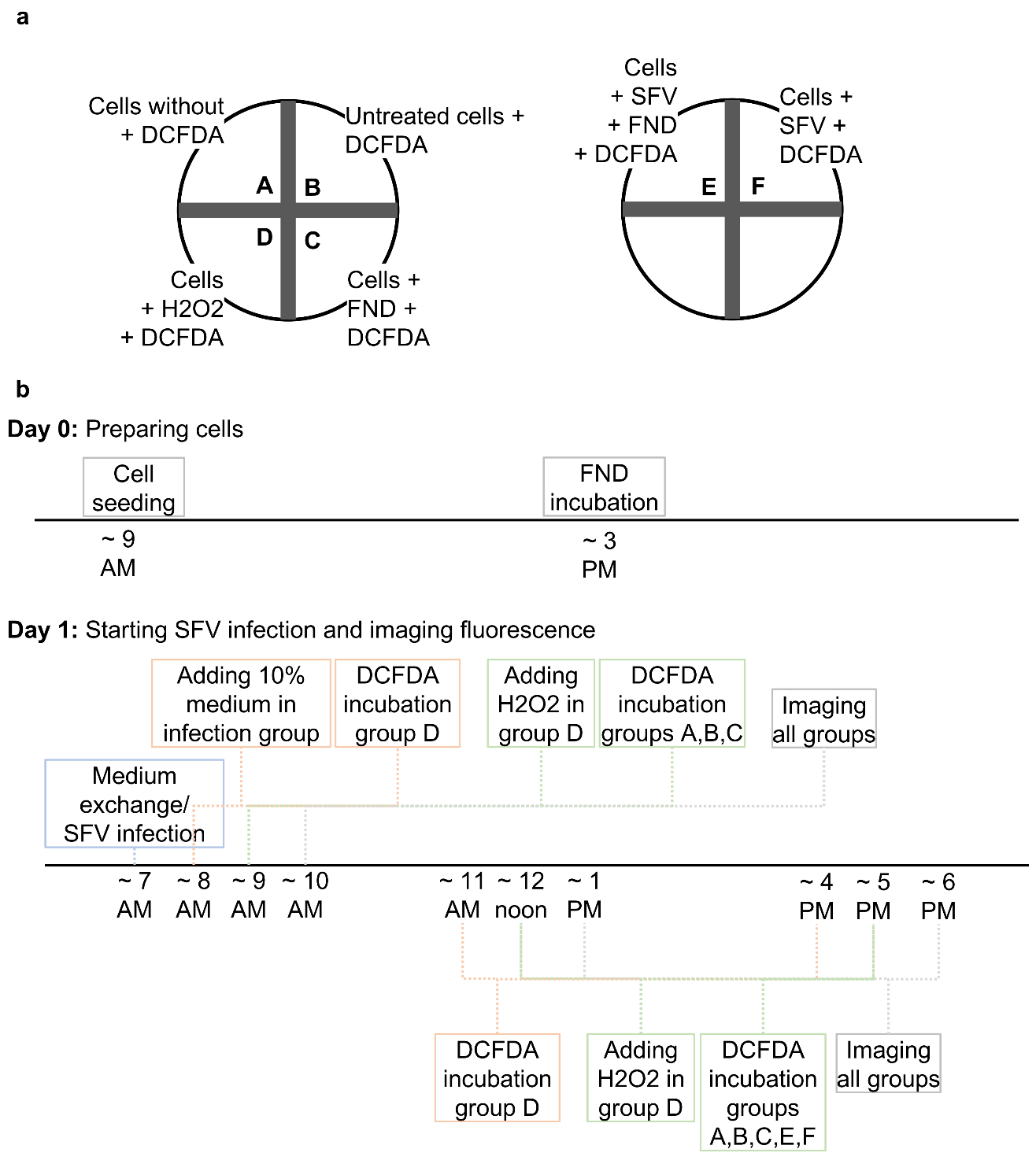


**Figure S6.** (a) Different groups used and (b) the timeline followed during the DCFDA ROS assay.

S8.2. Result and discussion


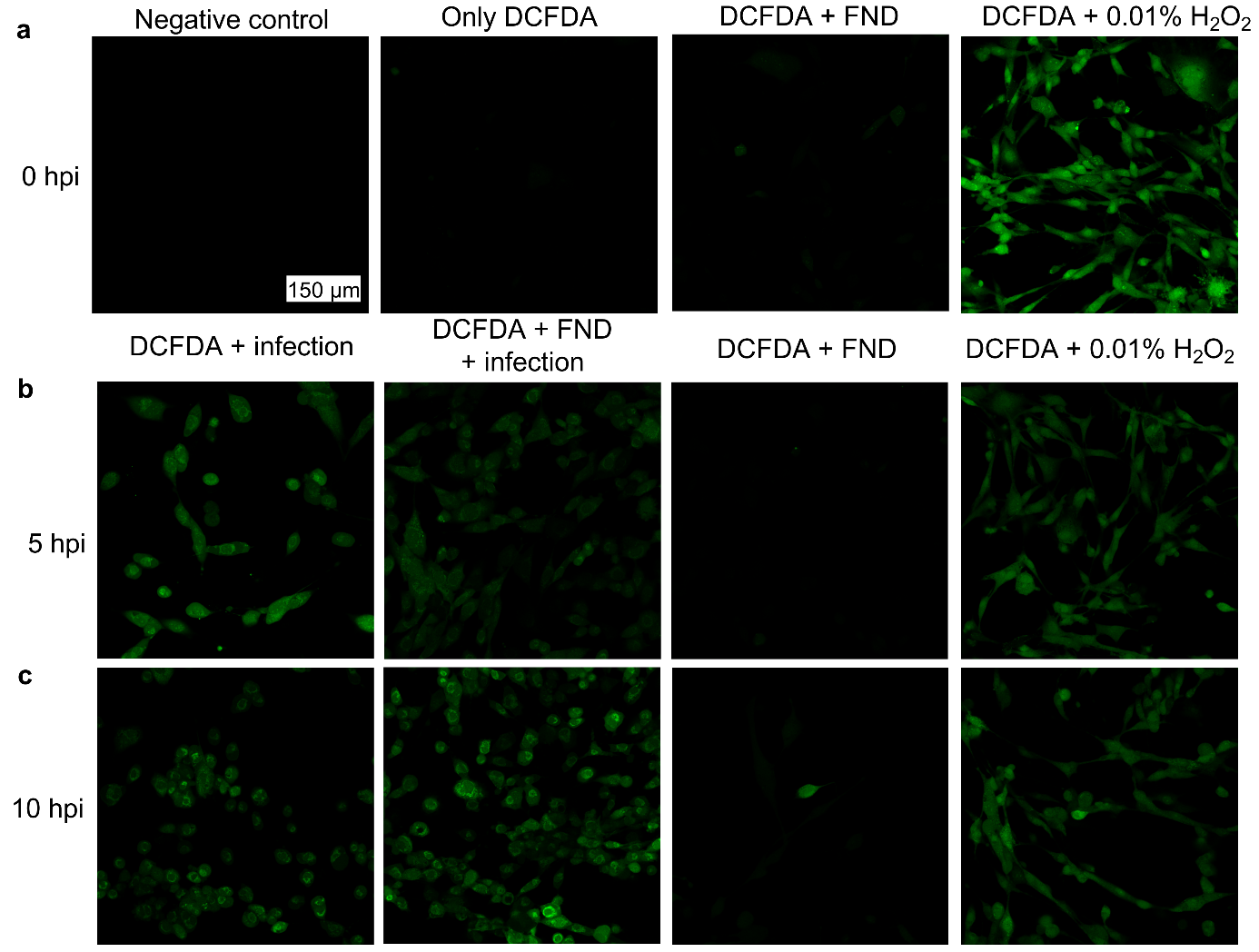


**Figure S7**. Results of fluorescence-based oxidative stress assay. Examples of images collected at (a) 0 hpi, (b) 5 hpi and (c) 10 hpi.

In this assay, non-fluorescent DCFDA molecules are converted into fluorescent molecules after reacting with ROS. Hence the amount of fluorescence is directly correlated with the amount of intracellular ROS. This assay measures the total amount of ROS produced within the cell during the incubation period whereas relaxometry specifically measures only the amount of free radicals at a given time. Although these two assays measure different entities, we performed the DCFDA assay to investigate the redox imbalance of the cells upon infection. We performed the DCFDA assay at 0, 5 and 10 hpi. At 0 hpi, untreated cells and cells having internalized FNDs did not show any fluorescence. This indicates that BHK-21 cells don’t trigger any ROS response upon internalizing FNDs.

Contrary to these two groups, almost all the cells in the positive control group, which were treated with 0.01% H_2_O_2_ showed bright fluorescence highlighting the oxidative stress caused by the H_2_O_2_. Results obtained for cells with internalized FNDs and positive control group at 5 and 10 hpi are very comparable to those corresponding groups at 0 hpi. However, the infected cells with and without FNDs show bright fluorescence at both 5 and 10 hpi, which is similar to the positive control. This indicates high degree of oxidative stress caused by the SFV infection.

**S8. Determining the optimal concentration of paraquat and N-acetylcysteine**

S8.1. Experimental methods

The optimal concentration of paraquat and N-acetylcysteine for experiments elaborated in **Section S6** was determined by analyzing the metabolic activity level of cells using an MTT assay. In the literature, Lin and coworkers reported that 250 µM paraquat induced intracellular superoxide production in the cells[6] whereas 0.1-80 mM N-acetylcysteine (NAC) was used for blocking oxidant production[7]. Using these concentrations as a reference, we explored a range of concentrations of these chemicals. Specifically, concentration of 50 µM, 100 µM, 250 µM, 500 µM, 750 µM, 1 mM, 2 mM, 5 mM, 7,5 mM, and 10 mM of paraquat were used. For N-acetylcysteine, we investigated the following concentrations- 0.1 mM, 0.5 mM, 1 mM, 2.5 mM, 5 mM, 10 mM, 25 mM, 50 mM and 75 mM. For every concentration we tested the metabolic activity of cells using an MTT assay.

For the MTT assay, 20,000 cells were plated in flat bottom 96-well plates followed by 24h of incubation at 37°C and 5% CO_2_. Then, the medium over cells was carefully removed and cells were washed with sterile PBS. Next, serum free cell media (RPMI media) were added into well plates followed by adding 5 mg/ml of MTT solution in sterile PBS. Cells were incubated with MTT solution for two hours at 37°C. After incubation, the liquid over cells from every well was collected and 2-propanol was added to dissolve the formazan. The plate was placed on a shaker for ~10-15 minutes. Photoabsorbance was read at 560 nm using a plate reader (Fluostar Optima).

S9.2. Results and discussion


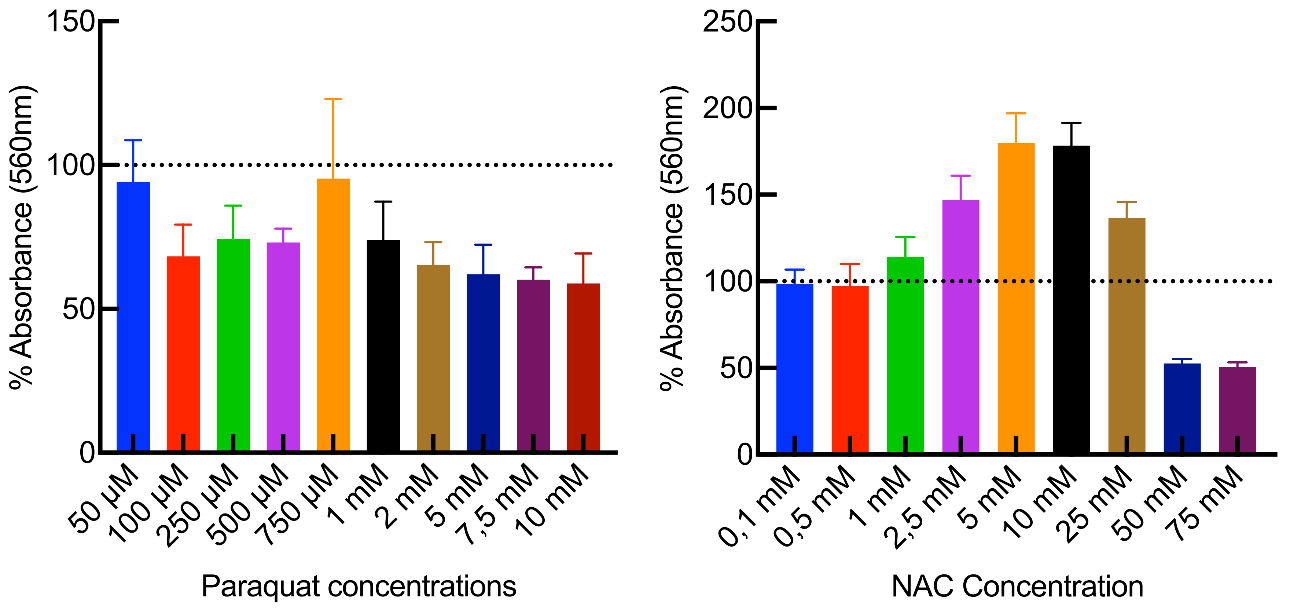


**Figure S8**. Absorbance was measured which is directly correlated with the cellular metabolic activity, when cells were treated with different Paraquat and NAC concentrations.

The amount of absorbance is directly coupled with the amount of formazan and hence with the metabolic activity of the cells. The concentration of Paraquat and NAC for which the metabolic activity of cells is altered the most compared to the control is determined to be optimal. This is in accordance with the rationale of the next experiment which is to assess the maximum change in the free radical composition under extreme conditions.

**S9. Free radical response of BHK-21 cells to oxidative/anti-oxidative stress by chemical treatments**

S9.1. Experimental methods

Here, we detected hydroxyl, superoxide and nitric oxide radicals using a commercially available fluorescent probes in three separate assays. Although these three assays were different from each other they had four basic steps- 1. Seeding 20,000 cells/well in a flat bottom 96-well plates (Corning Costar) followed by 24h of incubation at 37°C with 5% CO_2_. 2. Removing the old medium carefully and incubating cells with fluorescent probe suspension for one hour 3. Introducing cells to (i) 100 µM paraquat (ii) 5 mM N-acetylcysteine and (iii) 0.01% H_2_O_2_ for one hour incubation. (iv) Untreated cells and (v) cells incubated overnight with FNDs were also used as controls. 4. Washing cells with a buffer followed by measuring fluorescence intensity using a plate reader operating in bottom mode.

S9.1.1 Hydroxyl radical assay

A hydroxyl radical detection assay was conducted using a mitochondrial hydroxyl radical detection assay kit (Abcam, Netherlands). In the last step, cells were washed with warm sterile PBS and 100 µl assay buffer was added per well. Fluorescence intensity was recorded at 540(excitation)/590(emission) nm.

S9.1.2 Superoxide assay

This assay was performed using a superoxide detection assay (cell based) kit (Abcam, Netherland). 0.5 mM suspension of the probe was prepared using phenol red free RPMI media. Then the suspension was removed and cells were washed with washing buffer followed by adding 100 µl PBS buffer. Fluorescent intensity was measured at 495(excitation)/525(emission) nm.

S10.1.3 Nitric oxide assay

DAF-FM Diacetate (4-Amino-5-Methylamino-2',7'-Difluorofluorescein Diacetate) (Thermofisher, Netherland) was used for detecting nitric oxide formation. 5 µM probe in phenol red free RPMI medium was prepared. In step four, cells were washed with sterile PBS to remove excess medium. Then we replaced it with fresh phenol red free RPMI followed by 30 minutes incubation to allow complete de-esterification. Fluorescent intensity was measured at 495(excitation)/515(emission) nm.

S9.2. Results and discussion


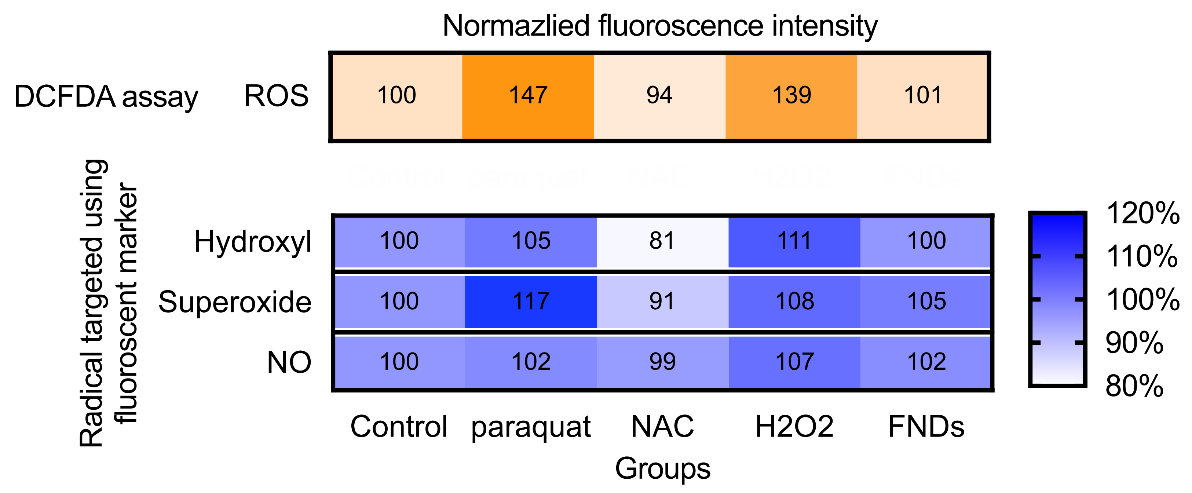


**Figure S9**. Heat map showing normalized fluorescence of DCFDA and free radical specific fluorescent markers quantified using a plate reader for different groups.

We gauged the maximum extent of the extra free radical production/nullification within BHK-21 cells upon introducing it to an extreme oxidative stress causing a external chemical stimuli or an anti-oxidant. Results shown in **Figure S11** indicate that, FNDs do not trigger a significant free radical production upon exposure to the chemicals. As expected, the superoxide-specific fluorescence probe intensity was maximal when the cells were treated with Paraquat. For the NAC treated cells, the fluorescence intensity was lower for all the free radical species. However, in none of the groups, the fluorescence intensity was higher or lower than 20% compared to the controls, suggesting that intracellular free radical amount do not dramatically fluctuate even after extreme oxidant/anti-oxidant chemical exposure. Interestingly, H_2_O_2_ which induces significant oxidative stress in cells does not seem to induce high concentration of free radicals. This indicates that the majority of the non-radical entities produced by a cell causing the oxidative stress in response to the H_2_O_2_ compared to non-paramagnetic species which do not yield any change in the T_1_ signal.

**S10.** **Schematic representation of the experimental scheme to investigate pseudo real-time variation in intracellular free radical amount post SFV infection**


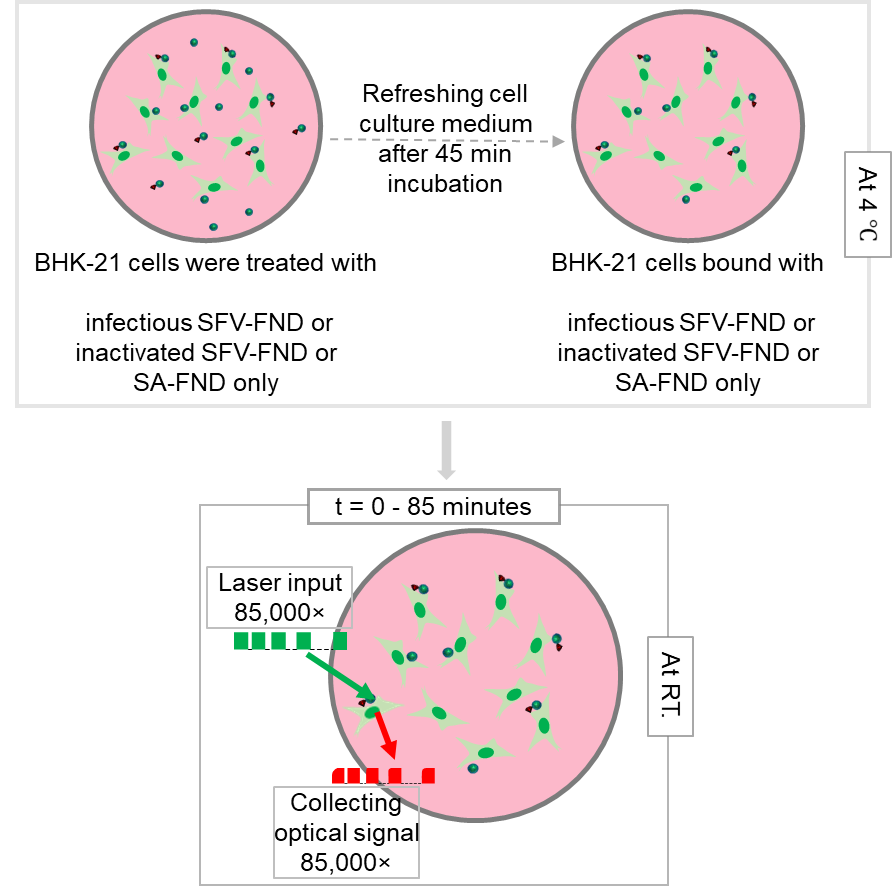


**Figure S10**. Schematic representation of the experimental scheme to investigate pseudo real-time radical variation using infectious SFV-FND, inactivated SFV-FND, and SA-FND.

**S11. Change in cellular morphology and cell viability as a function of time**


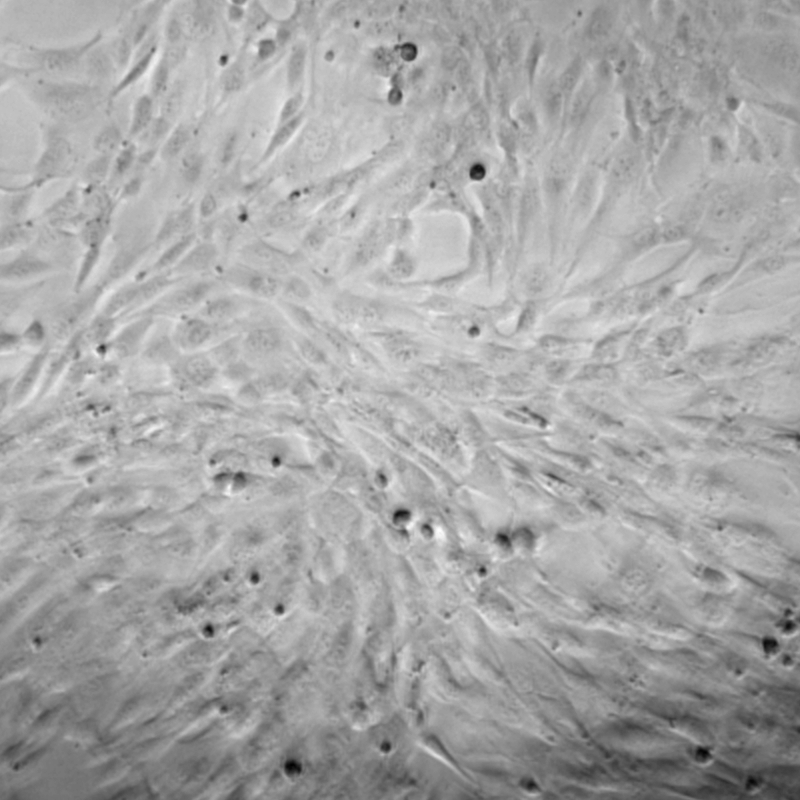

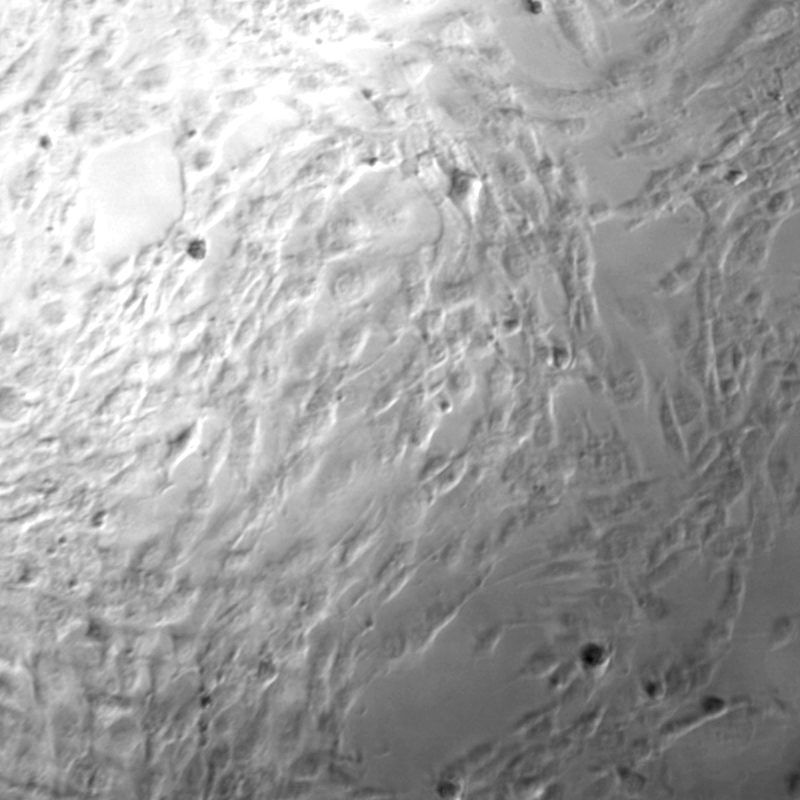

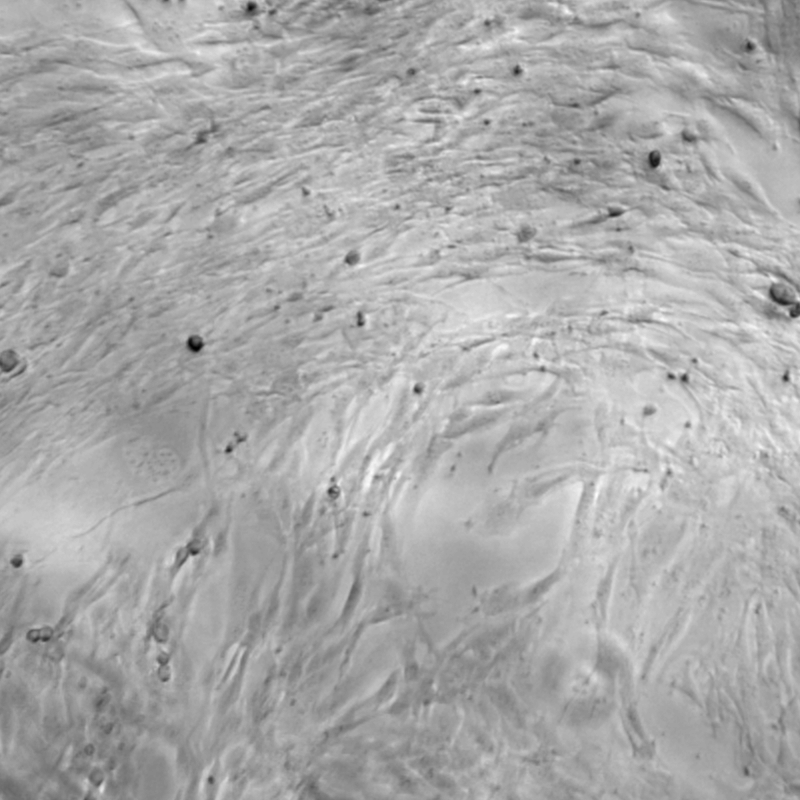

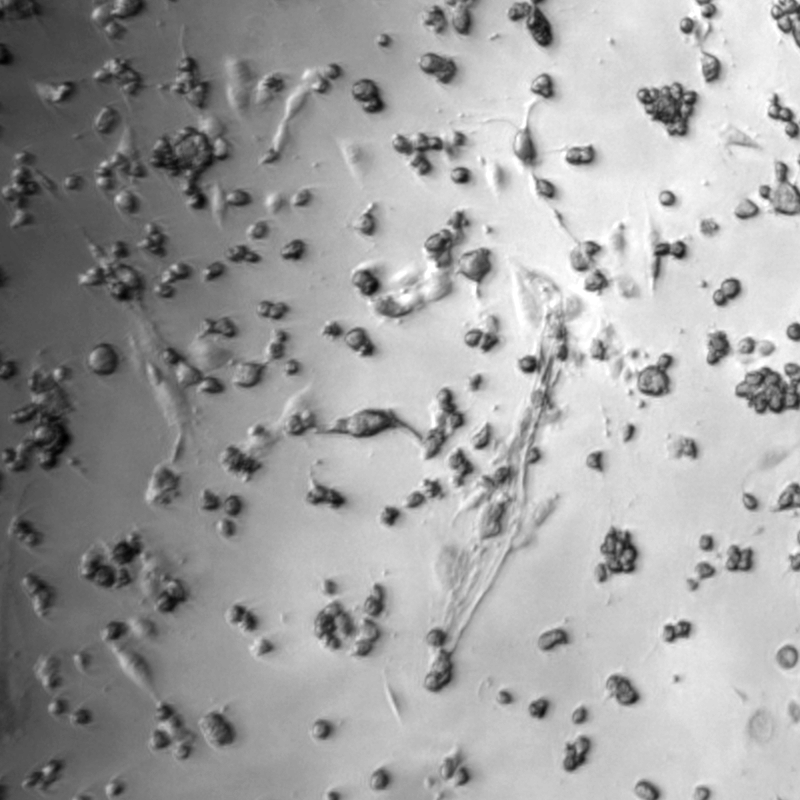


100 µm

SFV + BHK-21


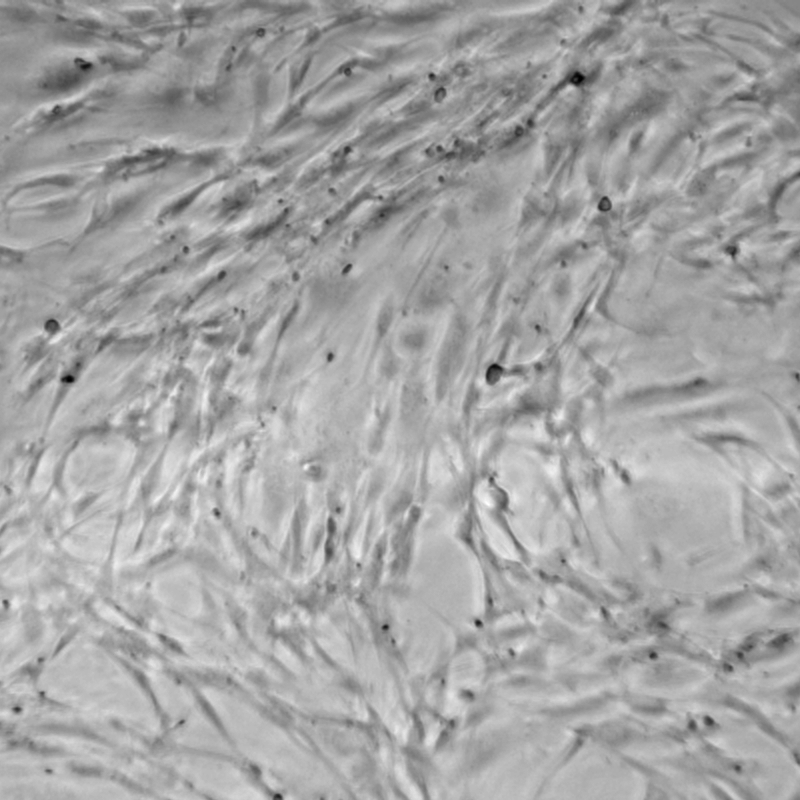

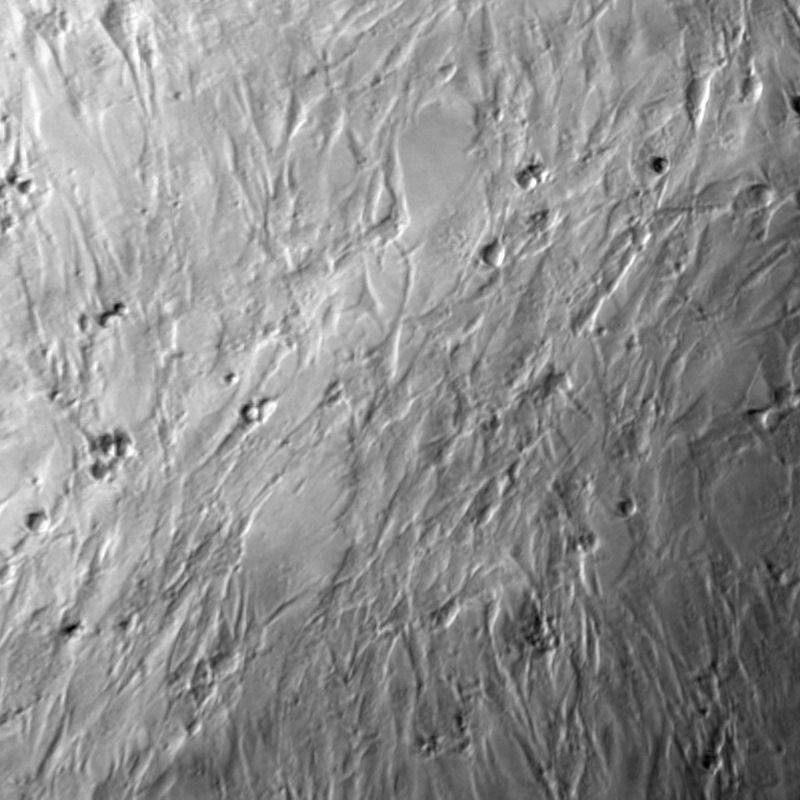

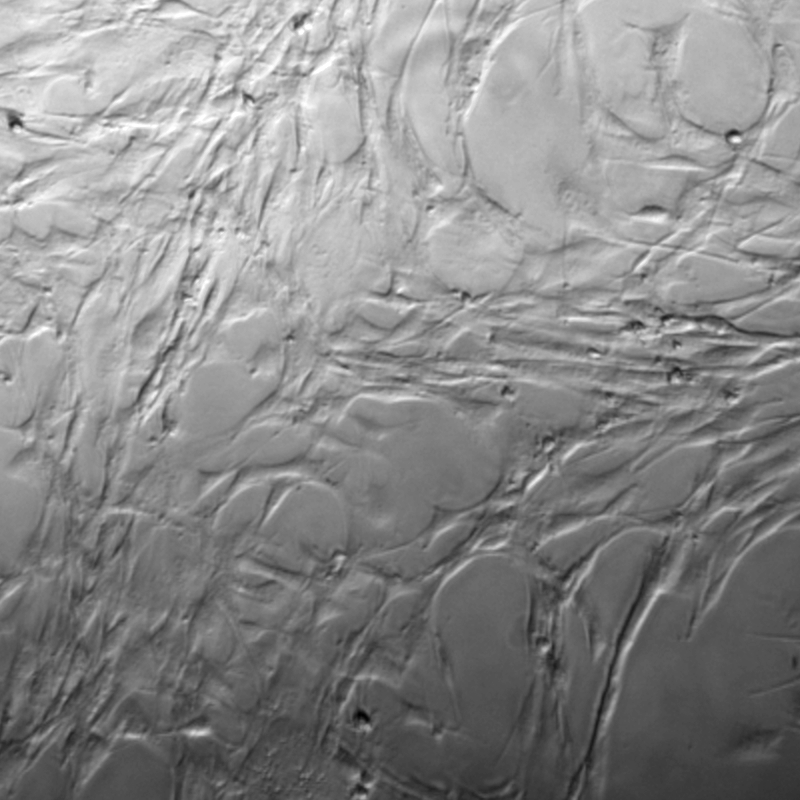

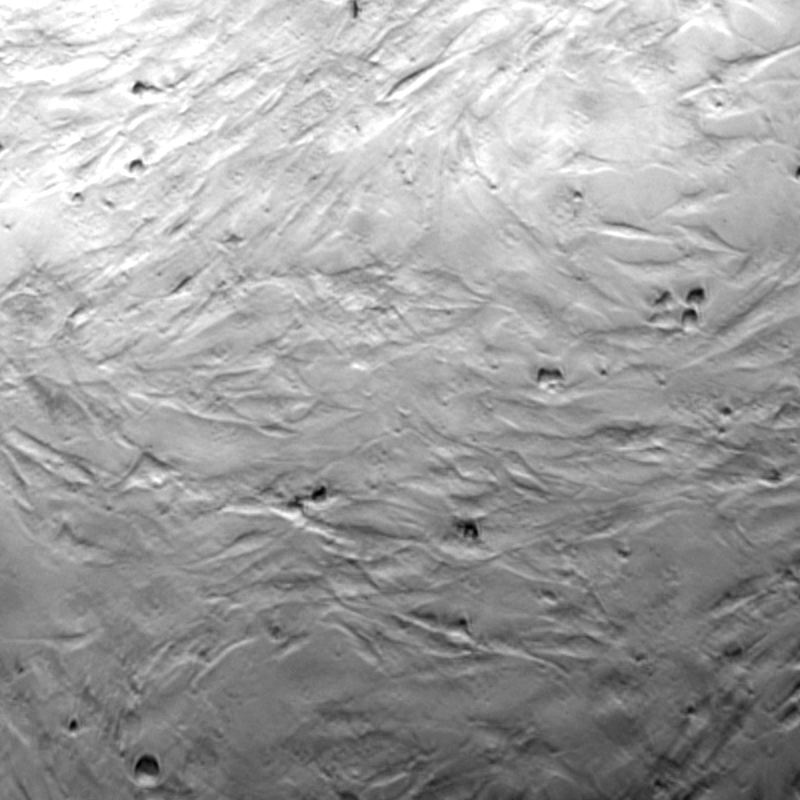


Inactivated SFV

+ BHK-21


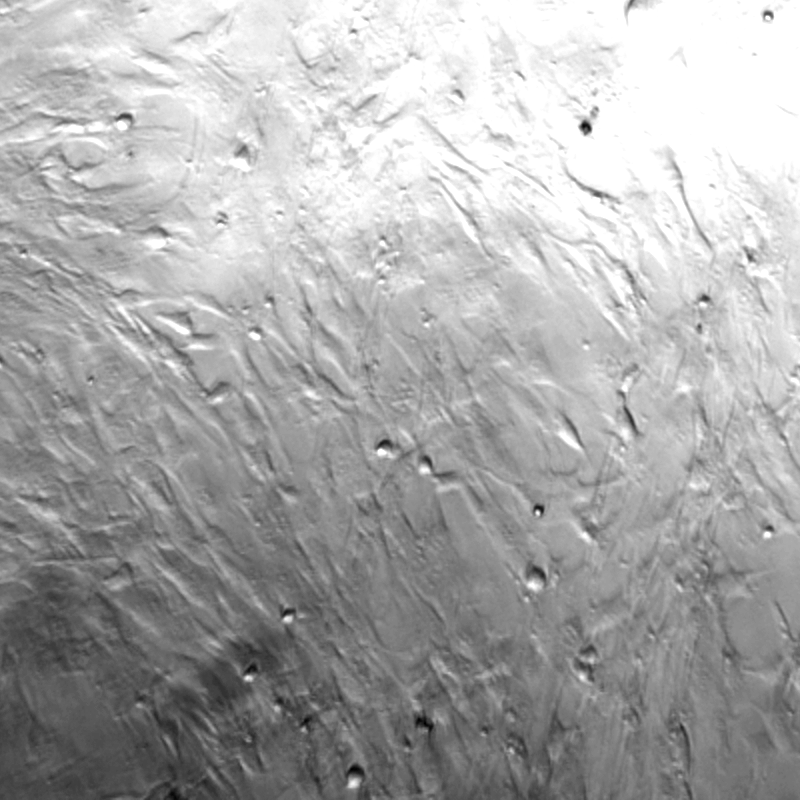

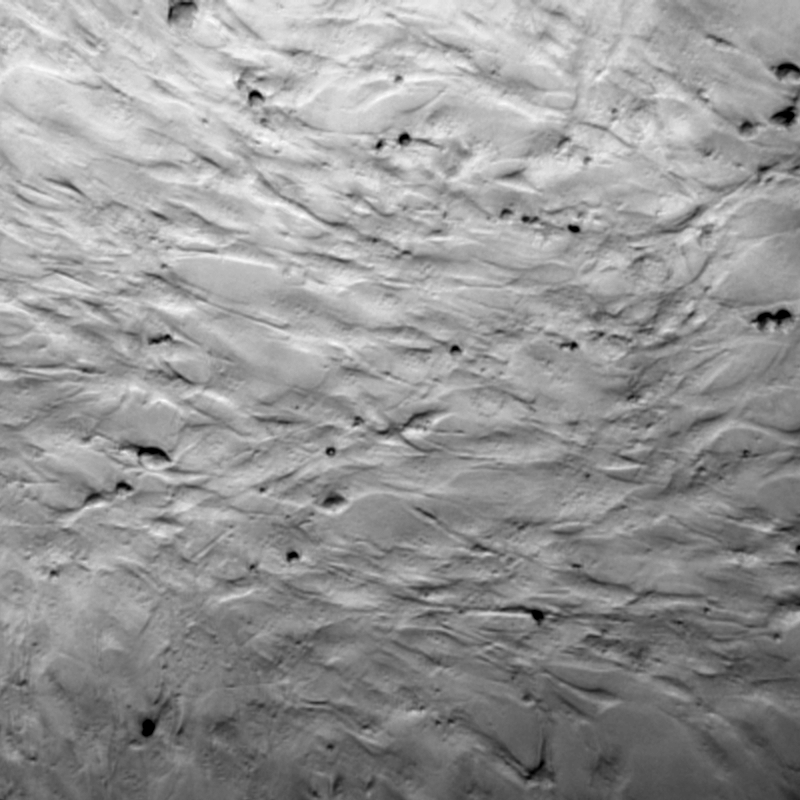

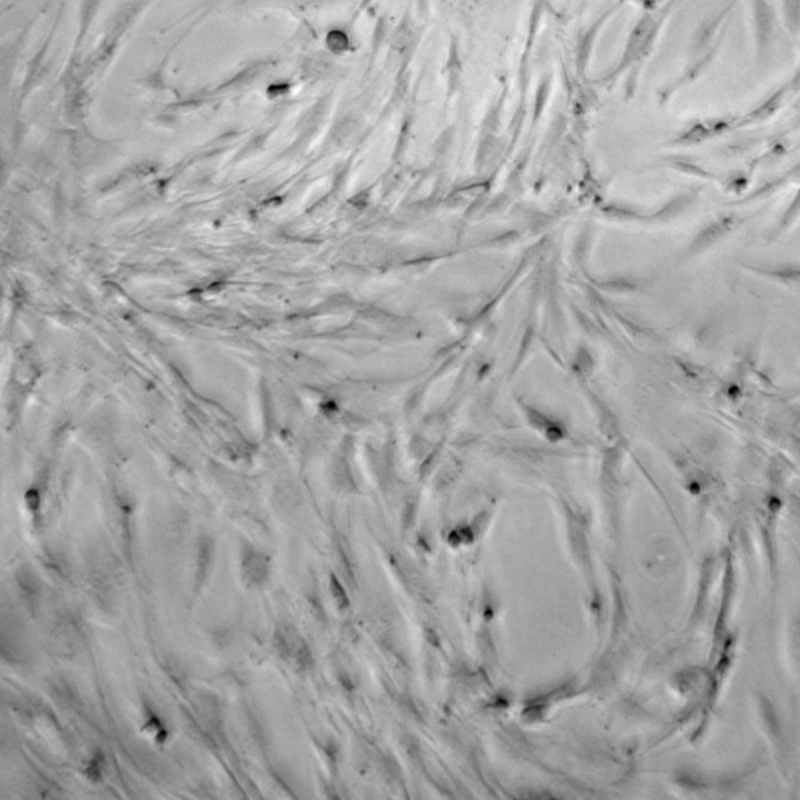

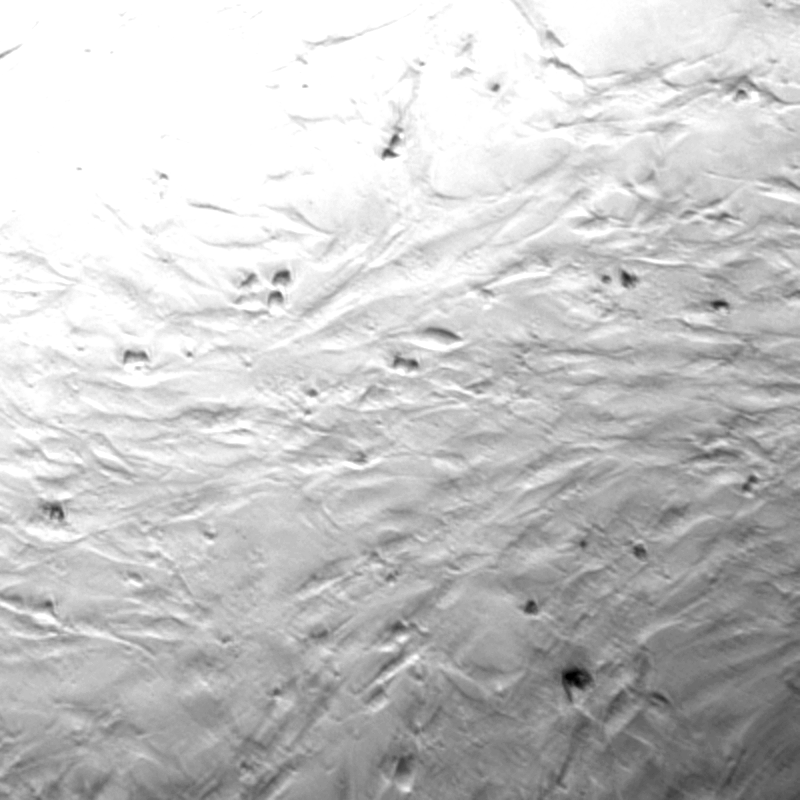


Untreated BHK-21

0 h

4 h

8 h

12 h

**a**

**b**

**c**

**d**

**Figure S11.** Images of SFV (a), inactivated SFV (b), and RPMI medium (c) treated BHK-21 cells at 0, 4, 8, and 12 hpi. (d) Cell viability of BHK-21 cells at different infection times.

The cell viability of BHK-21 during SFV infection was evaluated using an MTT assay at different infection times, by following the same infection procedure elaborated in the main manuscript. Specifically, SFV (MOI=20) and inactivated SFV were added to BHK-21 (10,000 cells/well) seeded in 96-well plates. After 0, 4, 8, and 12 hpi, the cells treated with live and inactivated SFV were imaged by differential interference contrast (DIC) microscope (Fig S10a-c). Then 3-(4,5-dimethylthiazol-2-yl)-2,5-diphenyl tetrazolium bromide (MTT) solution (50 μL, 5 mg/mL) in PBS was added to each well, and the cells were cultured for another 3 h. The MTT-formazan generated by BHK-21 cells was dissolved in 150 μL of DMSO, and the absorbance at 570 nm of each well was measured by a plate reader. The absorbance of the wells only containing DMSO was used as a background signal. The relative cell viability was determined by comparing the absorbance at 570 nm with cells cultured in RPMI medium (control group, without exposure to SFV or inactivated SFV). The data in **Figure S13d** mean ± SD (n=4).

The result of cell viability assay is consistent with the morphology study. In the first 8 h post infection by SFV, BHK-21 cells maintain their viability (above 95 %) and morphology. The MTT assay shows that the viability of cells treated with live SFV is around 50 % at 12 hpi, which can also be observed from the bright field images. Around 12 hpi, cells seem to start lysis after being infected by SFV (MOI=20). On the other hand, BHK-21 cells treated with inactivated SFV have similar cell viability and morphology with the control group even at 12 hpi.

**S12. Sensitivity of FND, SA-FND, and SFV-FND to GdCl_3_**

Two types of fluorescent nanodiamonds, 70 nm FND and streptavidin modified FND (SA-FND, 100 nm) were used in this work to investigate the intercellular radical response. In order to investigate the radical response near viral particles, SA-FND was further conjugated with SFV to form SFV-FND. A GdCl_3_ sensitivity experiment was performed to ensure that all kinds of FNDs used in this work (FND, SA-FND, and SFV-FND) show the same sensitivity to magnetic noise. Briefly, FND, SA-FND, and SFV-FND (1 µg FND equiv.) were seeded to 4-quartered glass-bottom petri dishes, respectively. Before the measurement, the GdCl_3_ solutions with desired concentrations (0, 1, 10…10^6^ nM) were added to dishes. T_1_ measurements were recorded on 5-6 diamond particles at each concentration according to the procedure described above. Figure S13 indicates that all the diamond particles and virus-diamond conjugates used in this work have a very similar sensitivity to magnetic noise, and the sensitive range is even less than 1nM Gd^3+^, which is more sensitive than conventional ESR.

**Figure S12**. Sensitivity of FND, SA-FND, and SFV-FND to GdCl_3_. Gd^3+^ was used as a contrast agent. The data was shown as mean ± SD (n=5-6).

References:

[1] H. Maeda, T. Akaike, Oxygen Free Radicals as Pathogenic Molecules in Viral Diseases. *Proc. Soc. Exp. Biol. Med.* 1991, *198* (2), 721-727. <https://doi.org/10.3181/00379727-198-43309C>.

[2] T. Akaike, H. Maeda, Nitric Oxide and Virus Infection. *Immunology.* 2000, *101* (3), 300-308. <https://doi.org/10.1046/j.1365-2567.2000.00142.x>.

[3] N. H. Acheson, I. Tamm, Replication of Semliki Forest Virus - an Electron Microscopic Study. *Virology.* 1967, *32* (1), 128-&. <https://doi.org/10.1016/0042-6822(67)90261-9>.

[4] L. Kaariainen, P. J. Gomatos, A Kinetic Analysis of Synthesis in Bhk 21 Cells of Rnas Specific for Semliki Forest Virus. *J. Gen. Virol.* 1969, *5*, 251-265. <https://doi.org/10.1099/0022-1317-5-2-251>.

[5] K. U. Dee, D. A. Hammer, M. L. Shuler, A Model of the Binding, Entry, Uncoating, and Rna-Synthesis of Semliki-Forest-Virus in Baby Hamster-Kidney (Bhk-21) Cells. *Biotechnol. Bioeng.* 1995, *46* (5), 485-496. <https://doi.org/10.1002/bit.260460513>.

[6] K. I. Lin, P. Pasinelli, R. H. Brown, J. M. Hardwick, R. R. Ratan, Decreased Intracellular Superoxide Levels Activate Sindbis Virus-Induced Apoptosis. *J. Cell Biol.* 1999, *274* (19), 13650-13655. <https://doi.org/10.1074/jbc.274.19.13650>.

[7] K. I. Lin, S. H. Lee, R. Narayanan, J. M. Baraban, J. M. Hardwick, R. R. Ratan, Thiol Agents and Bcl-2 Identify an Alphavirus-Induced Apoptotic Pathway That Requires Activation of the Transcription Factor Nf-Kappa-B. *J. Biol. Chem.* 1995, *131* (5), 1149-1161. <https://doi.org/10.1083/jcb.131.5.1149>.
